# Improved Zebra Optimization Algorithm with Multi Strategy Fusion and Its Application in Robot Path Planning

**DOI:** 10.3390/biomimetics10060354

**Published:** 2025-06-01

**Authors:** Zhengzong Wang, Xiantao Ye, Guolin Jiang, Yiru Yi

**Affiliations:** 1 School of Intelligent Manufacturing and Electronic Engineering, Wenzhou University of Technology, Wenzhou 325035, China; 2 Zhejiang Zhengli Enterprise Management Co., Ltd., Wenzhou 325035, China; 3 Ruian Security Group Co., Ltd., Wenzhou 325200, China

**Keywords:** zebra optimization algorithm, levy flight, path planning, engineering problems

## Abstract

In order to overcome the inherent drawbacks of the baseline Zebra Optimization Algorithm (ZOA) approach, such as its propensity for premature convergence and local optima trapping, this work creates a Multi-Strategy Enhanced Zebra Optimization Algorithm (MZOA). Three strategic changes are incorporated into the improved framework: triangular walk operators to balance localized exploitation and global exploration across optimization phases; Levy flight mechanisms to strengthen solution space traversal capabilities; and lens imaging inversion learning to improve population diversity and avoid local convergence stagnation. The enhanced solution accuracy of the MZOA over modern metaheuristics is empirically validated using the CEC2005 and CEC2017 benchmark suites. The proposed MZOA’s performance improved by 15.8% compared to the basic ZOA The algorithm’s practical effectiveness across a range of environmental difficulties is confirmed by extensive assessment in engineering optimization and robotic route planning scenarios. It routinely achieves optimal solutions in both simple and complicated setups. In robot path planning, the proposed MZOA reduces the movement path by 8.7% compared to the basic ZOA. These comprehensive evaluations establish the MZOA as a robust computational algorithm for complex optimization challenges, demonstrating enhanced convergence characteristics and operational reliability in synthetic and real-world applications.

## 1. Introduction

Metaheuristic algorithms, recognized as prominent bio-inspired computational frameworks within swarm intelligence paradigms, provide robust solutions for complex optimization problems through stochastic search mechanisms [1,2]. These algorithms provide self-adaptive optimization frameworks that may dynamically explore solution spaces by drawing theoretical underpinnings from natural processes (such as photosynthetic energy conversion) and biological systems (like collective animal behaviors) [3,4,5]. They represent a crucial area of artificial intelligence research because of their exceptional effectiveness in solving large-scale non-convex optimization problems, especially in high-dimensional search spaces, and because of their derivative-free nature, structural simplicity, and computational efficiency [6,7].

The metaheuristic category incorporates both traditional and newly emerging types. Well-established techniques consist of Particle Swarm Optimization (PSO) [8], Chicken Swarm Optimization (CSO) [9], Ant Colony Optimization (ACO) [10], Gorilla Troops Optimizer (GTO) [11], and Simulated Annealing (SA) [12]. In the latest developments, novel frameworks like Dung Beetle Optimizer (DBO) [13], Whale Optimization Algorithm (WOA) [14], Harris Hawks Optimization (HHO) [15], and Sparrow Search Algorithm (SSA) [16] have been introduced, along with other remarkable variants described in [17,18,19,20,21]. These optimizers, inspired by biological phenomena, have made substantial breakthroughs in various fields, such as computer vision, energy management systems, and engineering design optimization. Practical evidence indicates that continuous improvements in algorithms and progress in theoretical aspects will further boost their ability to deal with more and more intricate real-world optimization challenges.

Traditional swarm intelligence optimization algorithms generally face some common shortcomings and challenges when solving complex optimization problems. Firstly, they are prone to falling into local optima, especially when dealing with multimodal functions or high-dimensional space problems. The individuals in the algorithm are likely to prematurely converge and fail to explore the global optimal solution. Secondly, there is a dilemma in balancing the convergence speed and accuracy. Some algorithms may sacrifice the accuracy of solutions in pursuit of fast convergence, while improving accuracy often leads to a significant decrease in convergence speed. Thirdly, there is the problem of parameter sensitivity. The performance of the algorithm is highly dependent on the setting of initial parameters (such as population size, learning factors, inertia weight, etc.), and different parameter combinations may lead to significant differences in results, which increases the complexity of parameter tuning. In addition, when dealing with large-scale complex problems, the computational complexity of the algorithm increases sharply, resulting in low computational efficiency and difficulty in meeting real-time requirements. Finally, there is a weak theoretical foundation. Most swarm intelligence algorithms lack rigorous mathematical theoretical support, and the theoretical analysis of algorithm convergence, complexity, etc., is insufficient, which limits their application in high-reliability scenarios. The Zebra Optimization Algorithm (ZOA), put forward by Trojovská et al. in 2022 [22], is a metaheuristic framework inspired by biology. It mathematically formulates the dynamics of zebra herds via two fundamental survival mechanisms: foraging behaviors and predator evasion strategies. This optimizer, which does not rely on derivatives, exhibits advantages in terms of structural simplicity and operational efficiency during implementation and has been successfully applied in diverse multidisciplinary optimization fields [23]. Empirical verifications are as follows: Elymany et al. enhanced the maximum power point tracking accuracy through control architectures optimized by ZOA [24], Bui et al. improved renewable energy transmission planning using variants of ZOA [25], and Aydemir carried out pioneering research on multilevel threshold segmentation for neuro-oncological imaging [26].

However, similar to other metaheuristic approaches, ZOA demonstrates inherent limitations in global search capabilities and convergence behavior, particularly manifesting as premature convergence and local optima entrapment [27]. This limitation primarily originates from the algorithm’s operational mechanics: during foraging phases, collective migration toward dominant solutions induces excessive exploitation while constraining spatial exploration. Subsequent defense phases employ predator-specific adaptive defense mechanisms that generate controlled spatial dispersion, yet prove insufficient for establishing effective exploration–exploitation equilibrium. Consequently, population diversity progressively diminishes through iterative cycles, perpetuating local optima entrapment. These identified shortcomings necessitate systematic algorithmic enhancements focusing on exploration-exploitation balance optimization and extended domain adaptation studies [28].

In an attempt to overcome these constraints, recent methodological progressions have brought about improved variants of the ZOA. In [29], a modified ZOA was suggested, which integrated adaptive oscillatory weights and golden sine guidance mechanisms. The adaptive part dynamically adjusts the exploration–exploitation balance through nonlinear weight modulation. Meanwhile, the golden sine operator utilizes hyperbolic contraction dynamics to enlarge the initial search radii and improve the local search accuracy in the later stages. Nevertheless, this directional guidance mechanism shows a strong bias towards the solution space, which may potentially lead to being trapped in local optima. In parallel, as presented in [30], chaotic sine mapping was incorporated to increase the diversity of population initialization. This improvement led to faster convergence rates but had limited effectiveness in maintaining population diversity during the iterative steps. Especially in multimodal optimization scenarios, the modified algorithm was more likely to be trapped in local optima compared to current metaheuristics.

This research presents a Multi-Strategy Enhanced Zebra Optimization Algorithm (MZOA) to tackle the aforesaid limitations. The methodological novelties consist of three main elements: Firstly, a lens imaging opposition-based learning mechanism is introduced during the initial foraging stages. By leveraging the convex lens projection theory, it enhances population diversity and effectively broadens the exploration of the solution space. Secondly, triangular walking dynamics with randomly determined step sizes are employed to balance the exploration-exploitation trade-off. This approach accelerates the convergence rate while maintaining population heterogeneity. Thirdly, Levy flight operators are included in the defensive phases. They enable long-range random jumps, helping to avoid being trapped in local optima while still preserving solution precision.

Path planning is an NP-hard combinatorial optimization problem that involves finding the optimal trajectories between given coordinates. Current applications cover autonomous vehicle navigation systems, unmanned aerial vehicle trajectory optimization, and precision guidance systems. Traditional exhaustive search methods have unacceptably high computational complexity for real-time implementations. Intelligent optimization methods offer feasible alternatives by efficiently approximating Pareto-optimal solutions. Experimental verification shows that the MZOA has better performance metrics in path planning situations, especially in terms of convergence speed and solution accuracy when compared to benchmark algorithms.

The following are this paper’s primary contributions:

1. A multi-strategy augmented Zebra Optimization Algorithm (MZOA) is developed. Through the integration of Levy flight dynamics, triangular walk operators, and lens imaging opposition-based learning mechanisms, this framework demonstrates significant enhancement in global optimization efficacy.

2. The enhanced algorithm undergoes rigorous empirical validation through exhaustive experimental evaluations and comparative analysis.

3. Implementation in robotic path planning scenarios yields quantifiable performance metrics, demonstrating the algorithm’s practical engineering applicability.

This paper is organized as follows: Section 1 is the introduction, Section 2 introduces the Standard Zebra Optimization Algorithm, Section 3 introduces the improved Zebra Optimization Algorithm, Section 4 describes the improvement of the improved Zebra Optimization Algorithm, Section 5 presents the function performance test experiment and algorithm comparison, and Section 6 is the conclusion.

## 2. Standard Zebra Optimization Algorithm

The Zebra Optimization Algorithm (ZOA), introduced by Trojovská et al. (2022), addresses optimization challenges through mathematical modeling of zebra herd dynamics, specifically simulating anti-predation behavior and foraging strategies to achieve solution space exploration [31]. Within this framework, each zebra position encodes a candidate solution to the optimization problem. Comparative analyses demonstrate ZOA’s distinct advantages over conventional metaheuristic approaches, particularly in accelerated convergence rates and enhanced solution stability across 30-dimensional search spaces [32]. The algorithmic workflow comprises three principal phases: (1) Population initialization with opposition-based learning, (2) Foraging-phase collective intelligence optimization, and (3) Predator-response adaptive adjustment mechanisms.

### 2.1. Foraging Behavior

Update the population members’ locations by simulating zebra foraging behavior. One of these zebras, known as the pioneer zebra, directs other members of the population to more advantageous locations within the search space [33]. Consequently, the mathematical simulation used to update zebra locations throughout the foraging phase is

(1) $$x_{i,j}^{new,P1}=x_{i,j}+r\cdot \left(PZ_{j}-I\cdot x_{i,j}\right)$$

(2) $$X_{i}=\left\{\begin{array}{cc} X_{i}^{new,P1} & F_{i}^{new,P1}<F_{i}\\ X_{i} & \mathrm{else}\; \end{array}\right.$$

In the equation, $x_{i,j}^{new,P1}$ is the new state of the *i*th zebra in the *j*th dimension at the first stage, $x_{i,j}$ is the solution to the *j*th problem variable proposed by the *i*th zebra, and *r* is a random number within the interval [0,1]. *I* = round(1 + *r*), *I* ∈ {1,2}. ${\mathrm{PZ}}_{j}$ is the pioneer zebra in the *j*th dimension. $X_{i}^{new,P1}$ for the new state of the *i*th zebra in the first phase. Let $F_{i}$ be the objective function value, and $F_{i}^{new,P1}$ be the new objective function value for the first stage [34].

### 2.2. Defensive Behavior

Update the locations of population members in the search space by simulating the zebra’s defense mechanisms against predators. Depending on the predator, the zebra uses different protective techniques. The following circumstances are considered to occur with equal frequency in the ZOA design [35]:

(1) The S1 strategy is a mathematical model of the zebra’s escape strategy when it is attacked by a lion.

(2) The S2 strategy is used to mathematically describe the zebra’s offensive tactic when it is attacked by other predators.

Using Equation (4) to update the zebra’s position, the new position is acceptable if the zebra’s objective function value is higher there. This update condition’s mathematical modeling is

(3) $$X_{i,j}^{new,P2}=\left\{\begin{array}{cc} S_{1}:x_{i,j}+R\cdot (2r-1)\cdot (1-\frac{t}{T})\cdot x_{i,j} & P_{s}\leq 0.5\\ S_{2}:x_{i,j}+r\cdot (AZ_{j}-I\cdot x_{i,j}) & else \end{array}\right.$$

(4) $$X_{i}=\left\{\begin{array}{cc} X_{i}^{new,P2} & F_{i}^{new,P2}<F_{i}\\ X_{i} & else \end{array}\right.$$

In the formula, $X_{i,j}^{new,P2}$ represents the new state of the *i*th zebra in the *j*th dimension in the second phase, and $F_{i}^{new,P2}$ is the new objective function value in the second phase. *t* is the current iteration count, and *T* is the maximum iteration count. $P_{s}$ is the probability of choosing one of the two strategies, $P_{s}$ ∈ [0,1]. $AZ_{j}$ is the state of the attacked zebra, *R* is a constant equal to 0.01, and $X_{i}^{new,P2}$ is the new state of the *i*th zebra in the second stage [36].

## 3. Improved Zebra a Optimization Algorithm

### 3.1. Lens Imaging Reverse Learning Strategy

Opposition-based learning constitutes a computational enhancement mechanism that expands solution space exploration through inverse solution generation relative to current positions. This approach has demonstrated effectiveness in augmenting metaheuristic algorithm performance through solution diversity enhancement, as documented in [37,38,39]. However, the deterministic nature of conventional opposition-based solutions imposes inherent limitations: during local optima entrapment scenarios, the generated inverse solution may become suboptimal relative to current positions, failing to facilitate solution space escape. This critical limitation is effectively mitigated through lens imaging opposition-based learning mechanisms, which employ nonlinear projection principles to dynamically adjust solution space boundaries, as evidenced in recent studies [40,41].

**Figure 1:**
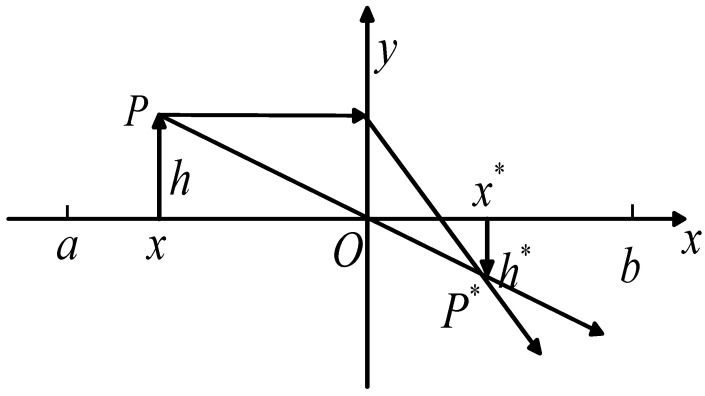
Opposition learning strategy based on lens image.

Figure 1 illustrates the lens imaging inverse learning technique. Using a two-dimensional space as an example, the convex lens is represented by the *y*-axis, and the search range for the solution is [*a*, *b*]. Assume that an object *P* has a height of *h* and that *x* is its projection on the *x*-axis. With a height of h* and a projection on the *x*-axis of x*, the item creates an inverted actual picture P* on the opposite side of the convex lens. In accordance with the convex lens’s image generation principle.

(5) $$\frac{(a+b)/2-x}{x^{*}-(a+b)/2}=\frac{h}{h^{*}}$$

Let $k=h/h^{*}$, then Equation (5) can be rewritten as:

(6) $$x^{*}=\frac{a+b}{2}+\frac{a+b}{2k}-\frac{x}{k}$$

Equation (6) is the solution formula for the inverse solution of the convex lens imaging inverse learning strategy. When *k* = 1, Equation (6) can be simplified to:

(7) $$x^{*}=a+b-x$$

This formula is the solution for reverse learning.

Reverse learning, which produces a fixed reverse solution, is a specific example of lens imaging reverse learning, as was previously noted. In lens reverse learning, a dynamically changing reverse solution may be generated by varying the size of *k*, which further improves the algorithm’s optimization potential. The following formula is used to obtain the *k* value in this paper:

(8) $$k={\left(1+{\left(\frac{t}{T}\right)}^{0.5}\right)}^{10}$$

### 3.2. Triangle Walking Strategy

The zebra will progressively look for food throughout the random walk phase, choosing a food site at random for position updates [42]. At this stage, it can move in a triangle pattern rather than straight approaching the meal. A random step length range is chosen as the walking direction once the distance between the food and a randomly selected reference agent has been determined [43]. Equations (9)–(14) are then used to determine the distance between the food and the point determined by the walk.

(9) $$L_{1}=PZ-X_{i}^{new,P1}$$

(10) $$L_{1}=PZ-X_{i}^{new,P1}$$

(11) β=2×π×rand()

(12) $$P=L_{1}^{2}+L_{2}^{2}-2\times L_{1}\times L_{2}\times \cos(\beta )$$

(13) $$r=rand()\times (3-(2*\frac{t}{T}))$$

(14) $$X_{i}^{new1,P1}=PZ+r\times P$$

Among them, *rand*() is a random number between [0,1], PZ is the pioneer zebra in the *j*th dimension, and $X_{i}^{new1,P1}$ is the new state of the *i*th zebra after performing a triangular walk in the first stage.

### 3.3. Levy Flight Strategy

This study presents the Levy flight approach to execute another position update in the search space, which successfully increases the diversity of zebra search and prevents the algorithm from entering local optima [44,45,46]. A random walk technique, the Levy flying strategy alternates between short and long distance movements [47,48,49]. The search range is wide when the step size is big, while local optimization is powerful when the step size is small [50]. The Levy flight strategy may be expressed as follows:

(15) $$levy=\frac{\mu }{{\left|\nu \right|}^{\frac{1}{\gamma }}}$$

In the equation, μ, ν follows a normal distribution, $\mu \sim N(0,\sigma_{\mu }^{2})$, $\nu \sim N(0,\sigma_{\nu }^{2})$, $\sigma_{\mu }^{2}$, and $\sigma_{\nu }^{2}$ are as in Equation (16):

(16) $$\left\{\begin{array}{c} \sigma_{\mu }={\left[\frac{\Gamma (1+\gamma )\sin(\gamma \pi /2)}{\gamma \Gamma \left((1+\gamma )/2\right)2^{(\gamma +1)/2}}\right]}^{1/\gamma }\\ \sigma_{\upsilon }=1 \end{array}\right.$$

Among them, γ is set to 1.5 [51,52].

After introducing the Levy flight strategy, the zebra’s position update formula is as follows:

(17) $$X_{i,j}^{new1,P2}=PZ+(PZ-X_{i,j}^{new,P2})*Levy$$

where $X_{i,j}^{new1,P2}$ is the new state of the *i*th zebra in the second phase after performing a Levy flight.

Prior to and following the Levy flying strategy update, compare the zebra placements. Perform the replacement procedure if the new position is superior; if not, keep the previous outcome. The main goal of implementing the Levy flying strategy is to improve the algorithm’s search capabilities by performing a second perturbation update based on the first location update.

### 3.4. The Pseudo Code of the Proposed MZOA

**Figure 2:**
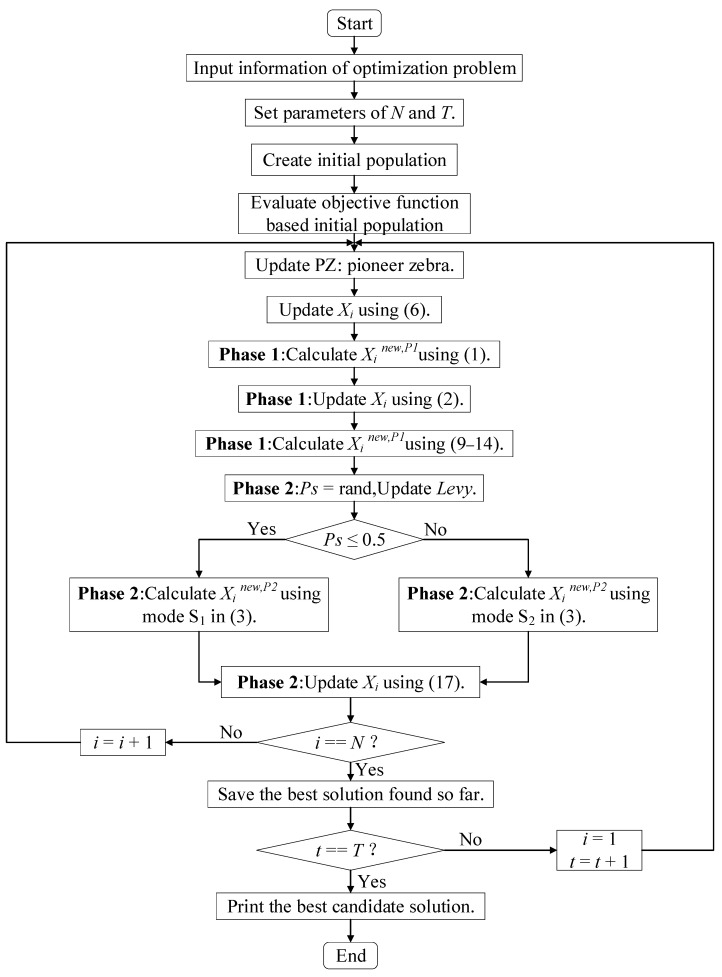
ASFFOX algorithm flowchart and pseudocode.

The pseudo code of the MZOA is as follows, with a comprehensive flowchart displayed in Figure 2: establish population *X*, compute fitness values, and initialize parameters *T*, *N*, *D*, etc.
**Algorithm 1:** Pseudo-Code of Proposed MZOA Start MZOA. 1.  Input: The optimization problem information. 2.  Set the number of iterations (*T*) and the number of zebras’ population (*N*). 3.  Initialization of the position of zebras and evaluation of the objective function. 4.    For *t* = 1: *T* 5.    Update pioneer zebra (*PZ*). 6.        Update the *i*th zebra using (6). 7.        For *i* = 1: *N*
8.            **Phase 1:** Foraging behavior 9.            Calculate new status of the *i*th zebra using (1). 10.          Update the *i*th zebra using (2). 11.          Calculate new status of the *i*th zebra using (9–14). 12.          **Phase 2:** Defense strategies against predators 13.          Update the *Ps*, *Levy*. 14.          If *Ps* < 0.5 15.              **Strategy 1**: against lion (exploitation phase) 16.              Calculate new status of the *i*th zebra using mode S1 in (3). 17.          else 18.              **Strategy 2:** against other predator (exploration phase) 19.              Calculate new status of the *i*th zebra using mode S2 in (3). 20.          end if 21.          Update the *i*th zebra using (17). 22.      end for *i* = 1: *N*
23.      Save best candidate solution so far. 24.  end for *t* = 1: *T*
25.  Output: The best solution obtained by MZOA for given optimization problem. End MZOA.

### 3.5. Algorithm Complexity Analysis

This study conducts a rigorous computational complexity analysis of both the Zebra Optimization Algorithm (ZOA) and its enhanced variant, the MZOA. The initialization phase of ZOA exhibits temporal complexity *O*(*N × D*), where *N* denotes population size and *D* represents problem dimensionality. During iterative optimization cycles, each candidate solution undergoes dual-phase updates with corresponding fitness evaluations, resulting in per-generation complexity *O*(*2N × D × T*), where *T* indicates maximum iteration count. Consequently, ZOA’s asymptotic complexity is formulated as *O*(*N × D ×* (*1 + 2T*)).

The enhanced MZOA maintains equivalent initialization complexity *O*(*N × D*) through equivalent population initialization procedures. Subsequent operational phases introduce three distinct computational components: lens imaging opposition-based learning during foraging initialization contributes *O*(*N × D × T*) complexity, triangular walk operators in foraging phases add *O*(*N × D × T*), while Levy flight integration in defense-avoidance phases further requires *O(N × D × T)*. The aggregate complexity *O*(*N × D ×* (*1 + 3T*)) demonstrates polynomial-time equivalence with baseline ZOA (*O*(*N × D ×* (*1 + 2T*)) *→ O*(*N × D × T*)), confirming that the multi-strategy enhancements preserve asymptotic computational efficiency while improving solution quality.

## 4. Simulation Experiment Analysis

### 4.1. Experiment and Environment Setup

To rigorously validate the optimization performance of the proposed MZOA, comprehensive benchmark function evaluations were conducted on both baseline and enhanced variants. A comparative analysis framework was established incorporating nine state-of-the-art metaheuristics: Whale Optimization Algorithm (WOA) [53], Harris Hawks Optimization (HHO) [54], Butterfly Optimization Algorithm (BOA) [55], Dung Beetle Optimizer (DBO) [56], Golden Jackal Optimization (GJO) [57], Spider Wasp Optimization (SWO) [58], Kepler Optimization Algorithm (KOA) [59], Subtraction-Average-Based Optimizer (SABO) [60], and the original Zebra Optimization Algorithm (ZOA) [61].

Experimental validation encompassed 30-dimensional benchmark functions from CEC2005 and CEC2017 test suites, supplemented by two real-world engineering challenges: mechanical design optimization and energy dispatch problems. Performance metrics including optimal solutions (minima), mean values, and standard deviations were systematically compared across all algorithms to quantify optimization efficacy and solution stability.

The experimental protocol employed rigorous comparative methodology, with each algorithm undergoing 30 independent trials under standardized conditions: population size fixed at 30 individuals and termination criterion of 1000 iterations. Parameter configurations for comparative algorithms strictly adhered to original literature specifications to ensure methodological consistency. Benchmark evaluation utilized CEC-standardized test functions spanning three categories: unimodal (exploitation assessment), multimodal (exploration capability), and fixed-dimensional multimodal (balanced evaluation) functions, with complete mathematical formulations and theoretical optima detailed in Table 1 and Table 2. All numerical experiments were executed on a Windows 11 platform using MATLAB R2020b computational environment, supported by an Intel Core i5-11400H processor (2.70 GHz) to ensure computational reproducibility.

### 4.2. Comparative Analysis of MZOA with Other Algorithms

**Figure 3:**
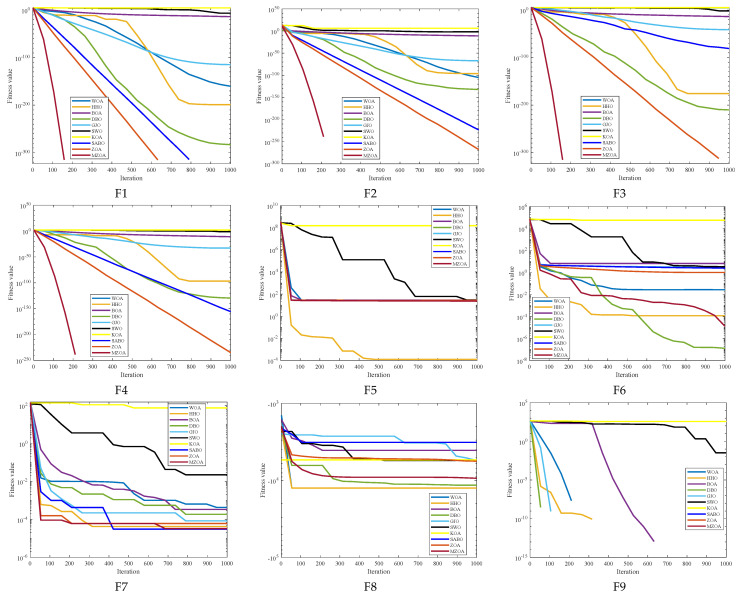

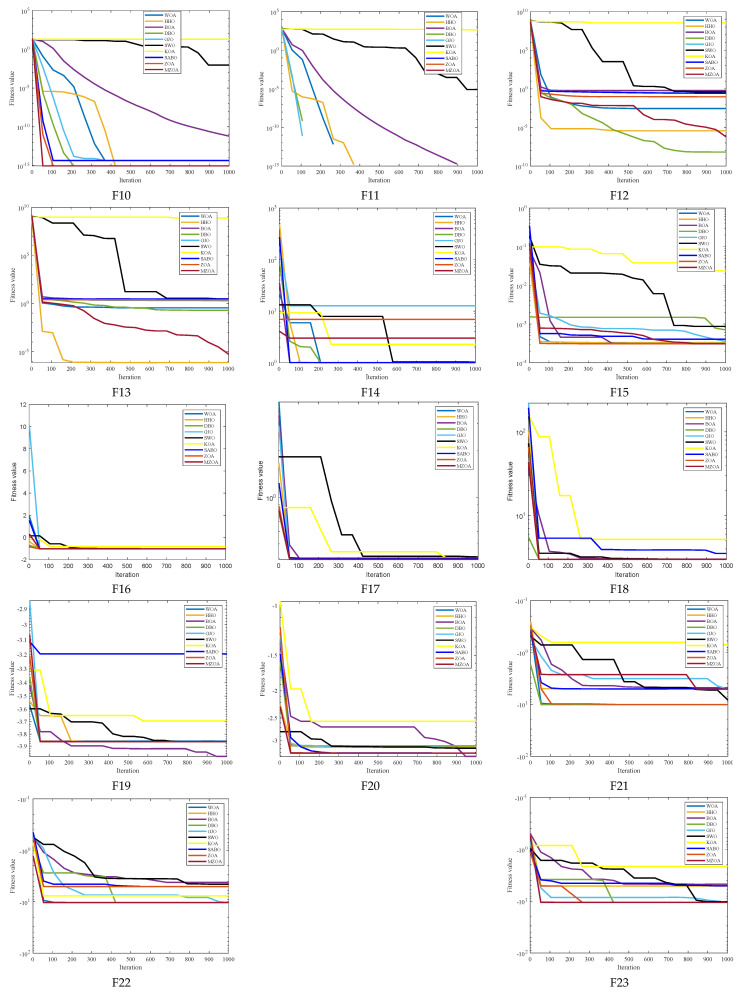
Iteration profile of CEC2005 of different improved algorithms.

**Figure 4:**
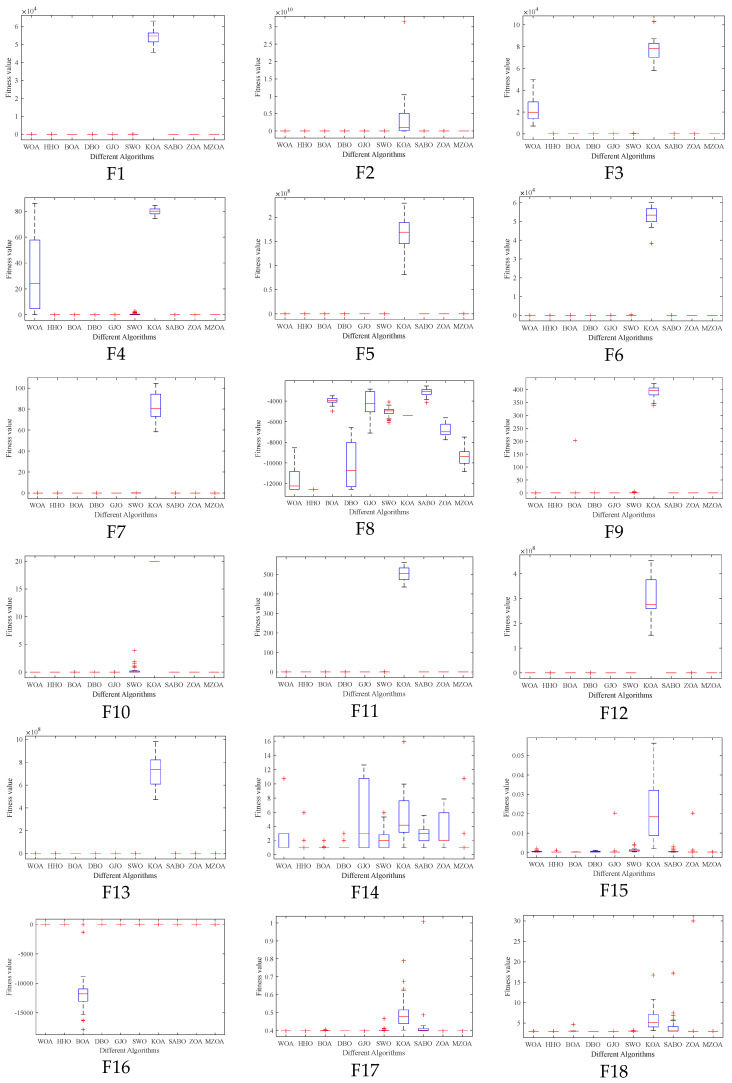

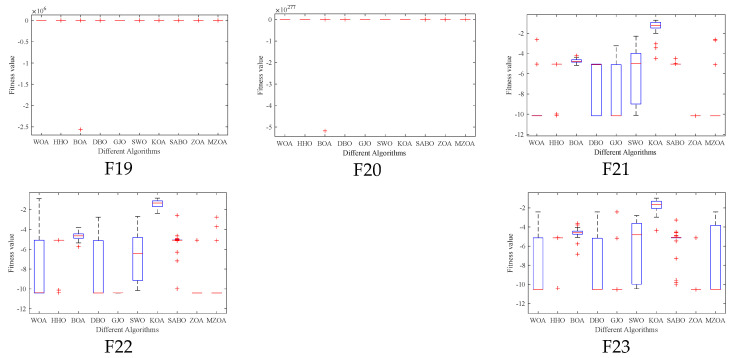
Box plots of CEC2005 of iterative data distributions.

**Figure 5:**
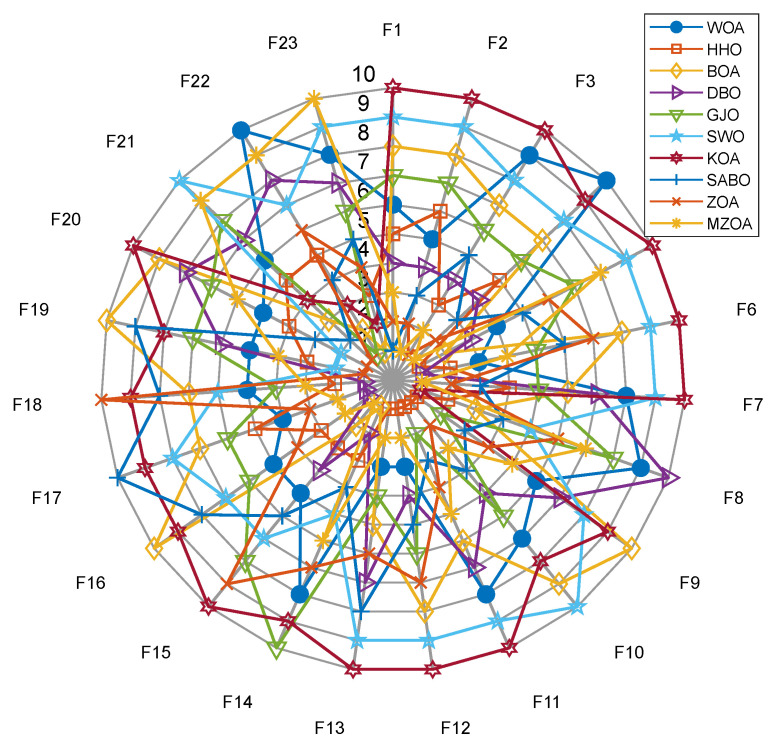
Data Radar Chart of CEC2005.

To rigorously validate the MZOA’s optimization efficacy, comprehensive benchmarking was conducted using CEC2005 standard test functions, comparing against state-of-the-art metaheuristics. Quantitative results from 30 independent trials are presented in Table 3, documenting three critical performance metrics: global minimum attainment (Min), mean fitness value (Avg), and solution stability (Std). Comparative analysis revealed the MZOA’s superior convergence characteristics through dual visualization modalities: Figure 3 illustrates the evolutionary convergence trajectory across iterations, while Figure 4 provides statistical distribution analysis via boxplot representations of final fitness values. Figure 5 shows the radar plots of 10 algorithms in different test functions.

Comprehensive analysis of the CEC2005 benchmark results reveals the enhanced MZOA’s superior optimization capability, demonstrating triple-criterion dominance (minimum, mean, and standard deviation) in 10 benchmark functions and achieving global optima in 17 test instances. For unimodal functions (F1–F4, F7), the MZOA exhibited absolute performance superiority across all evaluation metrics. In multimodal function optimization (F8–F13), the algorithm secured global optima in F14 while maintaining triple-criterion dominance in F9–F11. The composite function evaluations (F14–F23) further validated the MZOA’s robustness, with the algorithm attaining global optima in all cases except F15. Notably, in the F15 scenario, the MZOA still demonstrated statistical superiority through optimal mean and standard deviation values. These empirical findings collectively confirm the enhanced algorithm’s balanced exploration-exploitation characteristics and improved solution stability.

From the iteration curve in Figure 3, the box plot in Figure 4, and the data radar plot in Figure 5, we can intuitively understand the iteration results and data distribution. The MZOA demonstrates exceptional unimodal function optimization efficacy, particularly in F1-F4 benchmarks (Figure 3), reflecting enhanced exploration-exploitation capabilities crucial for global optimization in unimodal landscapes. This performance advantage originates from the algorithm’s balanced search strategy, where accelerated initial convergence (evident in steep early-stage gradients) transitions into dynamic stepwise refinement during later iterations, indicative of adaptive local optima avoidance mechanisms.

Boxplot analysis (Figure 4) reveals the MZOA’s superior solution stability through compact interquartile ranges and reduced outlier incidence. The consistent leftward skewness of solution distributions toward theoretical optima, coupled with a reduction in solution variance compared to the baseline ZOA, collectively validate the multi-strategy enhancement algorithm’s effectiveness in maintaining high-precision optimization while mitigating premature convergence risks. In the radar chart of Figure 5, it can be seen more clearly that the MZOA obtained better fitness values when solving most of the functions in the CEC2005 test function.

### 4.3. Further Comparative Experiments of the Algorithm

**Figure 6:**
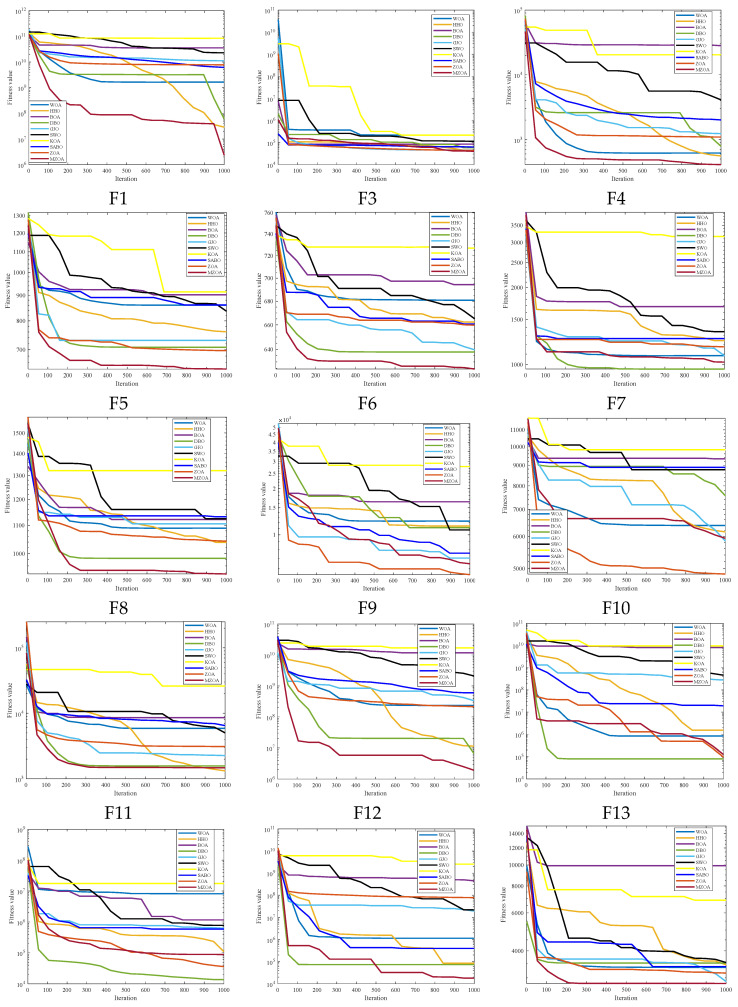

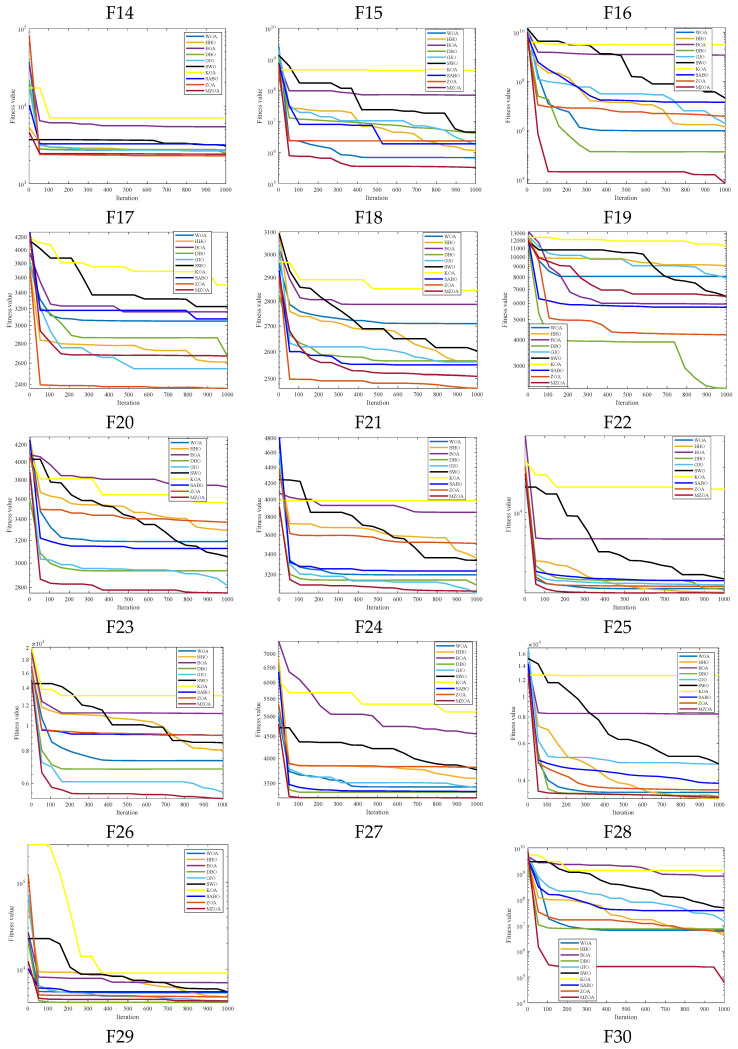
Iteration profile of CEC2017 of different improved algorithms.

To further validate the generalization capability and optimization efficacy of the proposed enhancement framework, extended benchmarking was conducted on the CEC2017 test suite 29 functions, excluding F2 due to numerical instability in MATLAB implementations). A comparative evaluation encompassing the MZOA, baseline ZOA, and eight state-of-the-art metaheuristics yielded comprehensive performance metrics documented in Table 4. This matrix presents critical optimization indicators: global minima attainment (min), mean fitness values (avg), and solution stability metrics (std), with optimal outcomes highlighted in boldface notation. Algorithm convergence dynamics are quantitatively characterized through Figure 5′s evolutionary trajectories, while Figure 6′s boxplot distributions statistically validate solution consistency across 30 independent trials. The experimental protocol maintained rigorous reproducibility standards through fixed random seeds and hardware-accelerated computation environments. Figure 5 shows the radar plots of 10 algorithms in different test functions.

Empirical analysis of CEC2017 benchmark results demonstrates the MZOA’s optimization limitations in five test functions (F13, F16–F18, F20) where global optima convergence was not achieved. However, the enhanced algorithm exhibits superior exploration–exploitation balance, attaining optimal mean fitness values in test cases (excluding F3, F10, F17, F20). This performance superiority stems from the synergistic integration of lens imaging opposition-based learning mechanisms and triangular walk operators, which collectively enhance solution space exploration capabilities. Although suboptimal in standard deviation metrics for the majority of the test functions, the MZOA demonstrates competitive solution stability. The algorithm’s consistent performance across multimodal landscapes (average rank 2 in Friedman test) confirms its robustness in maintaining exploration–exploitation equilibrium throughout iterative optimization processes.

The proposed MZOA demonstrates superior performance in attaining global optima for unimodal benchmarks (F1, F3) and multimodal functions (F4–F10), establishing its effectiveness in both exploitation and exploration paradigms. While exhibiting minor performance degradation in mean value metrics for F3 and F10 (relative to baseline ZOA), the algorithm maintains the quality of the solution within a small range of deviation, demonstrating strong potential for practical application despite its less-than-ideal standard deviation characteristics. In composite function evaluations (F21–F30), the MZOA achieves triple-criterion dominance (minimum value, average value, standard deviation) in the majority of test cases. Statistical analysis reveals non-significant differential margins in standard deviation metrics compared to state-of-the-art optimizers. These findings collectively validate the efficacy of the enhancement framework in restructuring the algorithm’s search dynamics, effectively balancing local optima avoidance with precision convergence capabilities through the strategic integration of Levy flight perturbations and opposition-based learning mechanisms.

**Figure 7:**
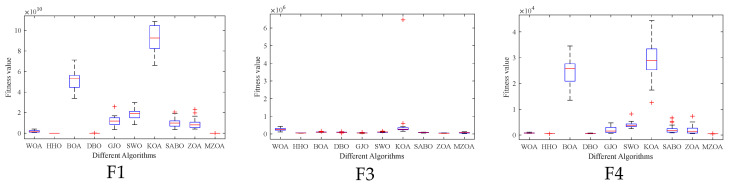

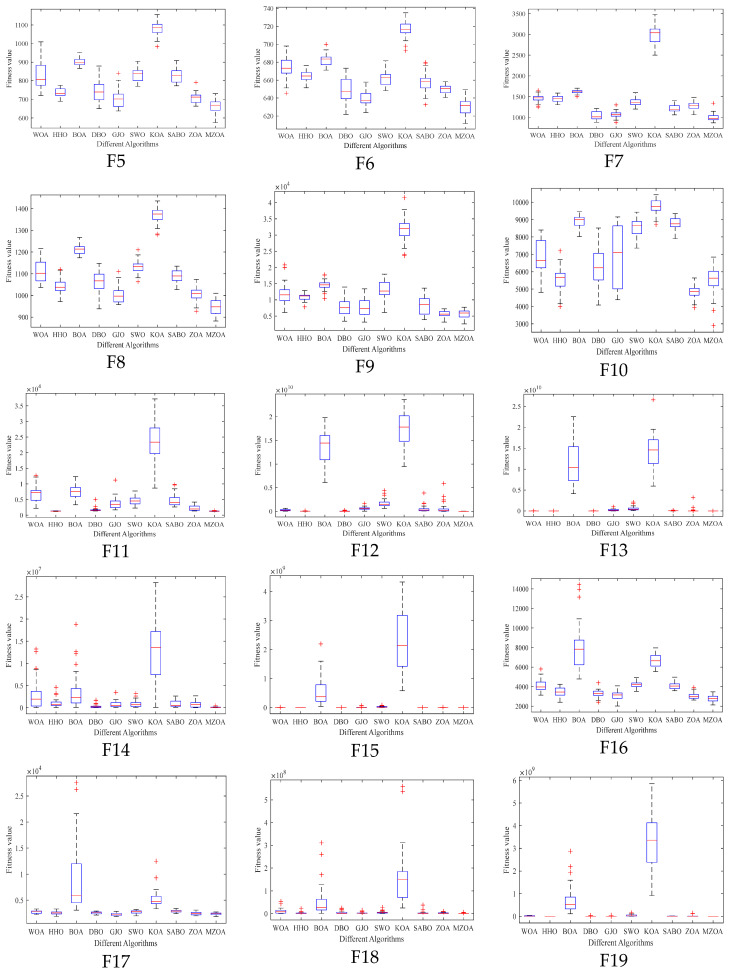

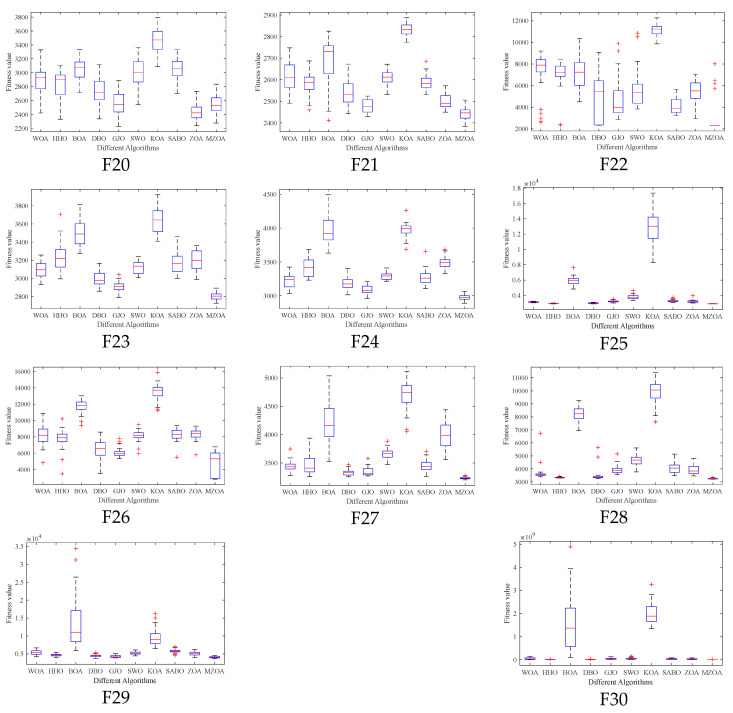
Box plots of CEC2017 of iterative data distributions.

**Figure 8:**
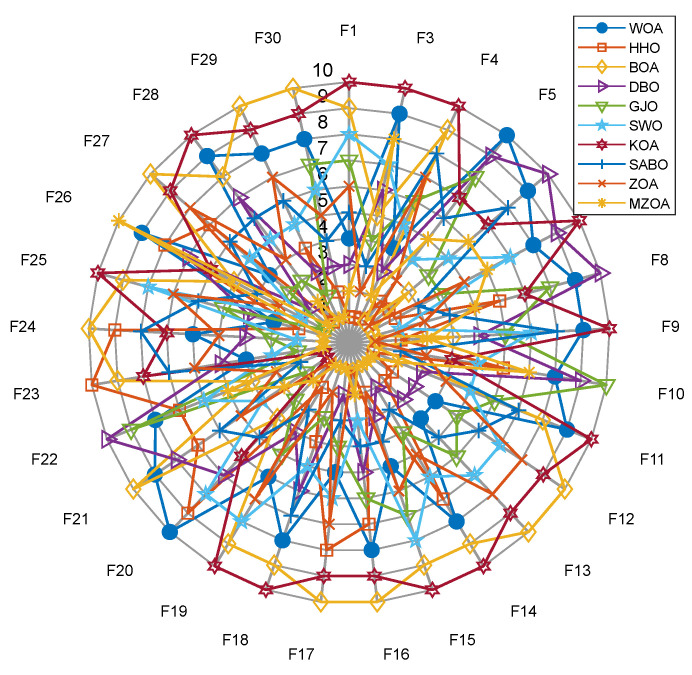
Data Radar Chart of CEC2017.

The convergence dynamics analysis in Figure 6 reveals the MZOA’s accelerated initial convergence characteristics, particularly within the first 100 iterations, demonstrating enhanced global search capabilities through triangular walk operators. This evolutionary trajectory transitions into adaptive refinement phases characterized by oscillatory convergence patterns, indicative of robust local optima circumvention mechanisms. Quantitative validation through Figure 7′s boxplot distributions elucidates the algorithm’s balanced performance profile: while solution stability metrics show non-significant deviation from comparative algorithms, the MZOA demonstrates superior statistical distribution characteristics through minimized outlier incidence and compact interquartile ranges. This performance equilibrium originates from the synergistic integration of Levy flight perturbations (maintaining population diversity) and lens imaging opposition-based learning (optimizing exploration-exploitation balance), collectively enabling sustained solution space exploration without compromising convergence precision. Combining the 10 algorithms in Figure 8 to solve the radar chart of the CEC2017 test set, it can be seen that the MZOA exhibits excellent fitness values in most test functions, proving that the improved ZOA has better solving accuracy.

### 4.4. Engineering Design Problem

#### 4.4.1. Tension/Compression Spring Design

Optimization of Engineering The optimization of a tensile/compression spring is the first problem. In order to satisfy the criteria of minimal deflection, vibration frequency, and shear stress, the problem seeks to design the spring dimensions in a reasonable manner that meet the requirements and have the least mass. The spring dimensions consist of the number of coils (*P*), the spring coil diameter (*d*), and the spring coil diameter (*D*). Three variables and four constraints are involved in this issue, which has the following mathematical description:

The goal function:

(18) $$Variable\;x=\left[d,D,P\right];$$

(19) $$\min f(x)=(x_{3}+2)x_{2}x_{1}^{2};$$

Constraints:

(20) $$g_{1}(x)=1-\frac{x_{3}x_{2}^{2}}{71,785x_{1}^{4}}\leq 0;$$

(21) $$g_{2}(x)=\frac{4x_{2}^{2}-x_{1}x_{2}}{12,566(x_{2}x_{1}^{3}-x_{1}^{4})}+\frac{1}{5108x_{1}^{2}}-1\leq 0;$$

(22) $$g_{3}(x)=1-\frac{140.45x_{1}}{x_{2}^{2}x_{3}}\leq 0;$$

(23) $$g_{4}(x)=\frac{x_{1}+x_{2}}{1.5}-1\leq 0;$$

Value range:

(24) $$0.05\leq x_{1}\leq 2;$$

(25) $$0.25\leq x_{2}\leq 1.3;$$

(26) $$2\leq x_{3}\leq 15;$$

**Figure 9:**
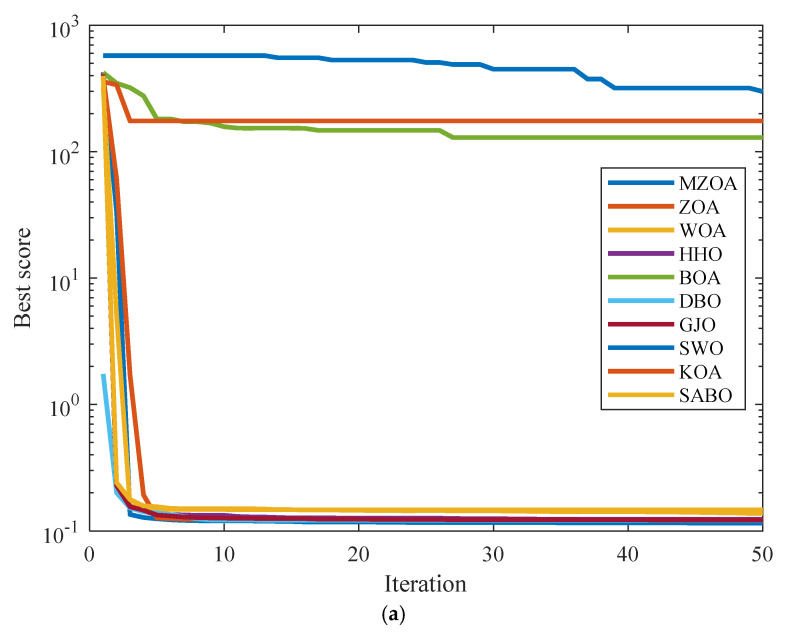

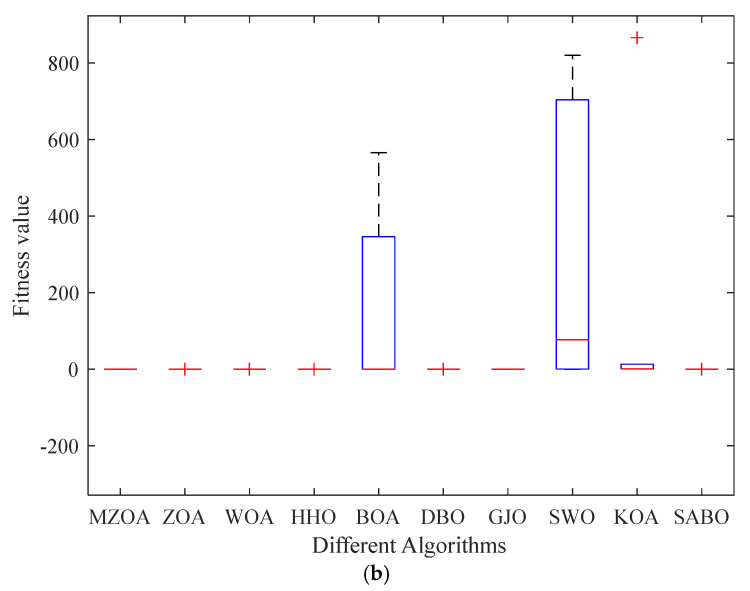
Iterative profile and box plots for extension/compression spring optimization problem. (**a**) Iteration curve graph. (**b**) Iterative data distribution box plot.

The comparative evaluation framework for Engineering Optimization Problem 1 incorporates the MZOA alongside nine established metaheuristics: the ZOA, WOA, HHO, BOA, DBO, GJO, SWO, KOA, and SABO. Experimental parameters were standardized with population size *N* = 30 and maximum function evaluations *T* = 50. Each algorithm underwent 10 independent trials, with complete optimization outcomes documented in Table 5. Analysis of the experimental data reveals the MZOA’s superior performance characteristics, achieving the minimum mean value and highest convergence precision among all evaluated algorithms. The algorithm’s third-ranked standard deviation further confirms its operational stability and consistent optimization capability. Convergence dynamics visualized in Figure 9 demonstrate the MZOA’s enhanced solution stability in spring design optimization, particularly evident in the steep gradient of initial convergence and sustained refinement in later phases. Boxplot distributions corroborate these findings through minimal solution variance and controlled outlier distribution patterns.

#### 4.4.2. Cantilever Beam

**Figure 10:**
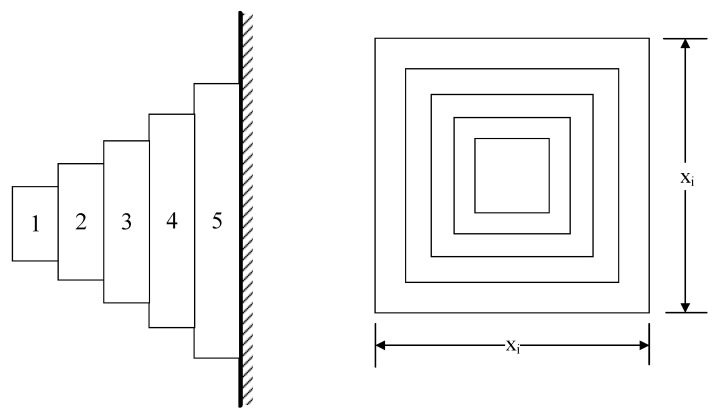
Schematic Diagram Of Cantilever Beam Design Problem.

Cantilever beam optimization is the second engineering optimization challenge. As seen in Figure 10, the structure of this civil engineering issue is made up of five hollow components, each of which has an identical thickness of hollow cross-section. Reducing or minimizing the cantilever beam’s weight is the aim. The cross-sectional widths of the five components are the design variables, while the beam’s thickness remains constant. In Equations (27) through (29), the mathematical model is shown.

The goal function:

(27) $$\min f(x)=0.0624(x_{1}+x_{2}+x_{3}+x_{4}+x_{5}),\;x_{i}>0$$

Constraints:

(28) $$g_{1}(x)=\frac{61}{x_{1}^{3}}+\frac{37}{x_{2}^{3}}+\frac{19}{x_{3}^{3}}+\frac{7}{x_{4}^{3}}+\frac{1}{x_{5}^{3}}\leq 1$$

Value range:

(29) $$0.01\leq x_{i}\leq 100,\;i=1,2,3,4,5$$

**Figure 11:**
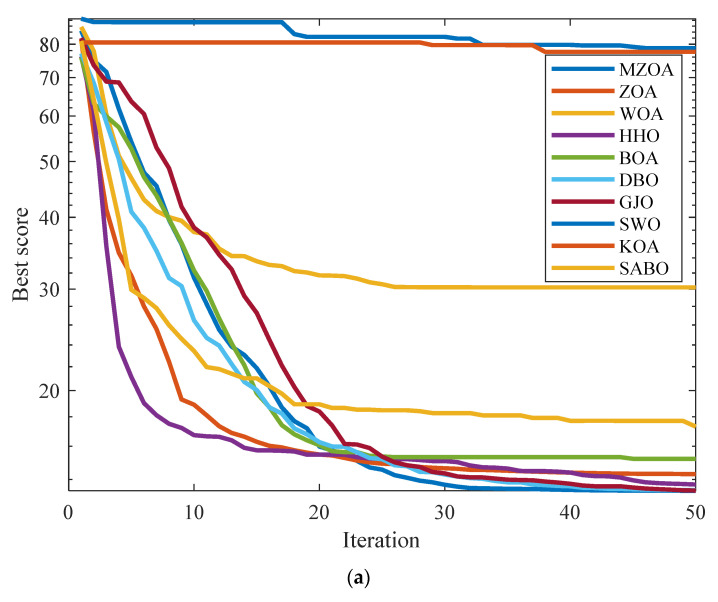

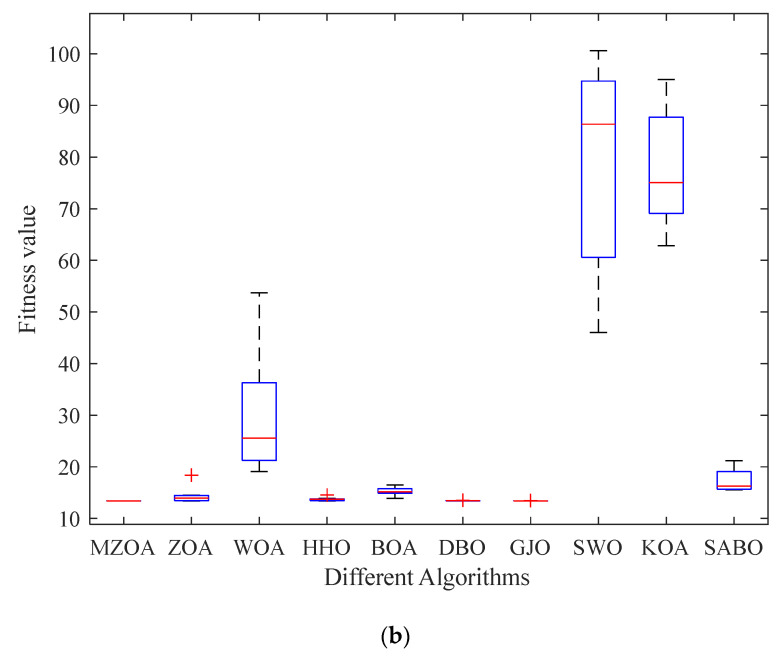
Iterative profile and box plots for cantilever beam design problem. (**a**) Iteration curve graph. (**b**) Iterative data distribution box plot.

For Engineering Optimization Problem 2, a comparative evaluation framework was established incorporating the MZOA with nine benchmark metaheuristics: ZOA, WOA, HHO, BOA, DBO, GJO, SWO, KOA, and SABO. The experimental protocol standardized population size (*N* = 30) and maximum function evaluations (*T* = 50), with each algorithm executing 10 independent trials. Comprehensive optimization results are documented in Table 6, complemented by convergence trajectory analysis in Figure 11 and corresponding boxplot distributions.

Empirical data analysis confirms the MZOA’s superior optimization performance in cantilever beam design challenges, attaining both the minimum mean value and competitive standard deviation metrics. This comparative advantage demonstrates the effectiveness of the multi-strategy enhancement framework in handling constrained engineering problems. Convergence characteristics reveal accelerated optimization dynamics during initial iterations followed by precision refinement phases, while boxplot distributions validate solution stability through compact interquartile ranges and controlled outlier dispersion patterns.

## 5. Robot Path Planning Problem

Path planning constitutes a critical NP-hard optimization problem in autonomous systems engineering, focusing on determining optimal trajectories between specified coordinates. Recent advancements in metaheuristic computation have witnessed growing adoption of swarm intelligence algorithms for optimal path generation tasks, demonstrating notable efficacy in complex navigation scenarios. To empirically validate the MZOA’s practical engineering utility, this investigation implements a comparative analysis framework incorporating ten state-of-the-art metaheuristics—the MZOA, ZOA, WOA, HHO, BOA, DBO, GJO, SWO, KOA, and SABO—applied to robotic trajectory optimization challenges. The experimental protocol systematically evaluates algorithmic performance in resolving real-world path planning constraints while maintaining computational tractability.

### 5.1. Path Planning Fitness Function

In the process of path planning, robots must be constrained by the following conditions: the robot must run within the grid map and cannot exceed the map boundary; The robot’s running path cannot pass through obstacles to avoid collisions; Robot path planning requires the shortest length to ensure obtaining the optimal path. Based on the above constraints, the fitness function for this problem is defined as:

(30) $$\min f=\sum_{i-1}^{n}L(i)+G(p_{i})$$

(31) $$L(i)=\sqrt{{\left(x_{i}-x_{i-1}\right)}^{2}+{\left(y_{i}-y_{i-1}\right)}^{2}}$$

(32) $$G(p_{i})=\left\{\begin{array}{cc} N*N & {\;P}_{i}\;\mathrm{is}\;\mathrm{not}\;\mathrm{passable}\\ 0 & {\;P}_{i}\;\mathrm{is}\;\mathrm{passable} \end{array}\right.$$

In the formula: *i* is the number of iterations; *L*(*i*) is the path planning length of the robot calculated by Euclidean distance during the *i*th iteration; (*x_i_*, *y_i_*) are the coordinates of the robot at the *i*-th iteration; *G* (*p_i_*) represents the judgment of whether the path point has exceeded the boundary or crossed obstacles during the *i*th iteration. If so, return the larger constant *N* × *N*, otherwise return 0.

### 5.2. Path Planning Environment Setup

**Figure 12:**
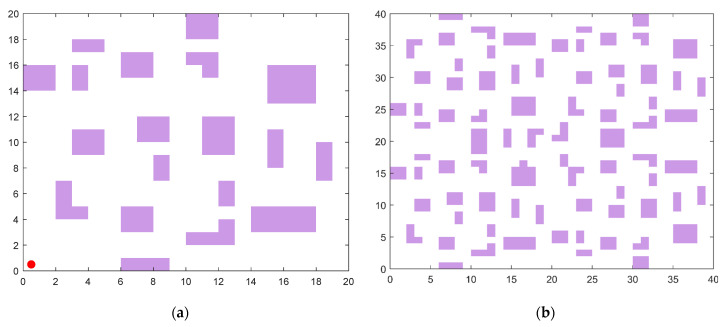
Grid map. (**a**) 20 × 20. (**b**) 40 × 40.

The robotic operational environment is formalized within a two-dimensional Cartesian coordinate system, employing grid decomposition methodology for spatial discretization. In a grid map, purple cells represent obstacles in the actual environment, while white cells indicate passable paths. As illustrated in Figure 12, two discretized environmental topologies were constructed with differential complexity scales: 20 × 20 and 40 × 40 grid configurations. The purple area is the obstacle, and the red dot is the starting point.

The experimental protocol defines the origin coordinate (0, 0) as initial configuration and the terminal coordinate (*n*, *n*) as target position, where n denotes grid matrix dimensionality. The mobile agent is modeled as a kinematic point-mass entity, while obstacle geometries are represented through Minkowski sum expansion to ensure collision-free trajectory planning under non-holonomic constraints. These environmental configurations serve as standardized testbeds for comparative pathfinding analyses. 

### 5.3. Robot Path Planning Simulation and Result Analysis

The experimental protocol standardized critical parameters across comparative scenarios: maximum iteration count *T* = 100 and population size *N* = 50 for all ten algorithms, ensuring methodological consistency between low-complexity (20 × 20) and high-complexity (40 × 40) environments. Quantitative outcomes from 10 independent trials are systematically compared in Table 7 through three principal metrics: global optima attainment, arithmetic mean, and solution stability indices (standard deviation).

**Figure 13:**
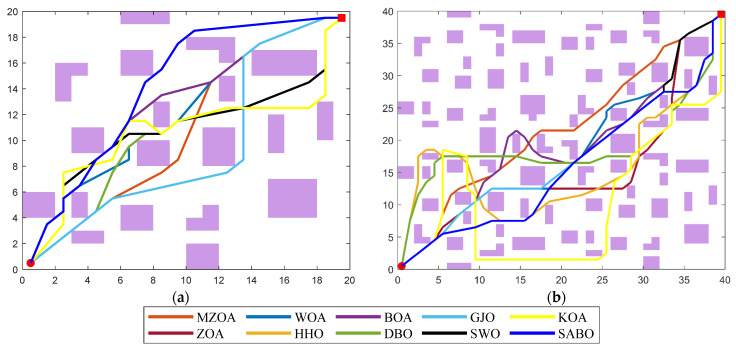
Comparison of path planning at the different sizes. (**a**) 20 × 20. (**b**) 40 × 40.

**Figure 14:**
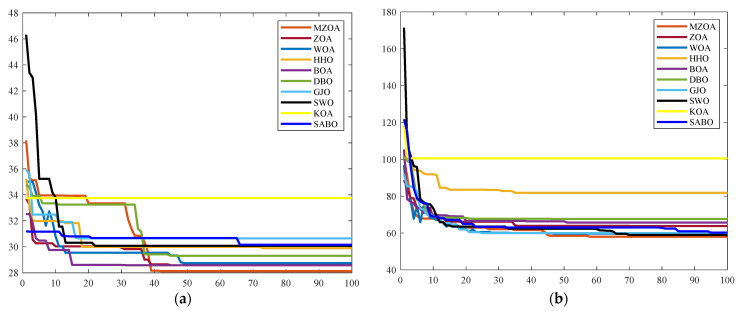
Comparison of iterative curves for algorithms of different sizes. (**a**) 20 × 20. (**b**) 40 × 40.

Algorithmic performance characteristics are further elucidated through dual visualization modalities: The path planning diagrams obtained by the algorithm in two types of maps are shown in Figure 13, and the iteration curve diagram is shown in Figure 14. These comparative visualizations collectively reveal the MZOA’s enhanced capability in maintaining geometric feasibility while navigating through constrained environments, particularly evident in complex obstacle-dense configurations.

Comparative analysis of trajectory optimization outcomes (Figure 13) demonstrates the MZOA’s superior trajectory smoothness, characterized by minimal waypoint transitions and optimized curvature continuity. The algorithm’s obstacle negotiation strategy maintains sufficient safety margins through vertex-proximal navigation, adhering to Minkowski sum-based collision avoidance principles. This geometric optimization capability ensures compliance with kinodynamic constraints while minimizing path tortuosity.

Convergence characteristics in Figure 14 reveal the MZOA’s nonlinear convergence dynamics, exhibiting multi-stage fitness improvement patterns across both environmental configurations. The algorithm’s adaptive phase-switching mechanism enables periodic solution space reinitialization, effectively circumventing local optima stagnation. The empirical validation conducted through the data in Table 7 confirms that the MZOA identified minimum path lengths of 28.130 and 56.584 in the two maps, respectively. These operational characteristics substantiate the enhancement algorithm’s efficacy in real-world navigation scenarios requiring precision and reliability.

## 6. Conclusions

This research puts forward a Multi-Strategy Enhanced Zebra Optimization Algorithm (MZOA) to deal with the original ZOA’s vulnerability to being trapped in local optima. The enhancement structure combines three mechanisms inspired by biological phenomena: Firstly, during the population initialization stage, a lens imaging opposition-based learning mechanism is utilized. By applying the convex lens projection principles, it increases the diversity of the solution space. Secondly, in the foraging phases, triangular walk operators are added. These operators allow for the modulation of stochastic step-sizes, enhancing the unpredictability of exploration through non-Euclidean movement patterns. Finally, Levy flight operators are incorporated into the modeling of defensive behavior. They generate scale-free search trajectories that have a probability of escaping local attraction basins while maintaining the solution precision. Empirical assessments show that the MZOA framework has better optimization effectiveness across two different engineering optimization scenarios and traditional benchmark test suites. A comparative analysis reveals statistically notable improvements in avoiding local optima and in convergence precision compared to current metaheuristics, thus validating its usability in complex engineering design fields. In robotic path planning applications, compared to the baseline ZOA implementations, the MZOA managed to reduce the trajectory length by 1.59% in a 20 × 20 grid and 9.05% in a 40 × 40 grid. These quantitative improvements, along with a decrease in computational load, confirm the algorithm’s enhanced operational efficiency in autonomous navigation systems.

Despite these advancements, there are still limitations in terms of stability in high-dimensional search spaces. Future research will focus on two investigation directions: (1) expanding its application to multidisciplinary scientific fields through the development of hybrid architectures; (2) creating stability-enhancement mechanisms by means of adaptive parameter tuning while still maintaining the Pareto-optimal convergence characteristics.

## Figures and Tables

**Table 1:** CEC 2005 partial test functions.

	No.	Functions	*Fi = Fi*(*x*)
	F1	Sphere Function	0
	F2	Schwefel’s Problem 2.22	0
Unimodal	F3	Schwefel’s Problem 1.2	0
Functions	F4	Schwefel’s Problem 2.21	0
	F5	Generalized Rosenbrock’s Function	0
	F6	Step Function	0
	F7	Quartic Function, i.e., Noise	0
	F8	Generalized Schwefel’s Problem 2.26	−12,569.5
Simple	F9	Generalized Rastrigin’s Function	0
Multimodal	F10	Ackley’s Function	0
Functions	F11	Generalized Griewank’s Function	0
	F12	Generalized Penalized Function 1	0
	F13	Generalized Penalized Function 2	0
	F14	Shekel’s Foxholes Function	0.9980
	F15	Kowalik’s Function	0.0003075
	F16	Six-Hump Camel-Back Function	−1.0316
	F17	Branin Function	0.3979
Composition	F18	Goldstein-Price Function	2.99999999
Functions	F19	Hartman’s Family	−3.8628
	F20	Hartman’s Family	−3.3220
	F21	Shekel’s Family	−10.1532
	F22	Shekel’s Family	−10.4029
	F23	Shekel’s Family	−10.5363

**Table 2:** CEC 2017 partial test functions.

	No.	Functions	*Fi = Fi*(*x*)
Unimodal Functions	1	Shifted and Rotated Bent Cigar Function	100
	2	Shifted and Rotated Sum of Different Power Function	200
	3	Shifted and Rotated Zakharov Function	300
Simple Multimodal Functions	4	Shifted and Rotated Rosenbrock’s Function	400
	5	Shifted and Rotated Rastrigin’s Function	500
	6	Shifted and Rotated Expanded Scaffer’s F6 Function	600
	7	Shifted and Rotated Lunacek Bi_Rastrigin Function	700
	8	Shifted and Rotated Non-Continuous Rastrigin’s Function	800
	9	Shifted and Rotated Levy Function	900
	10	Shifted and Rotated Schwefel’s Function	1000
Hybrid Functions	11	Hybrid Function 1 (*N* = 3)	1100
	12	Hybrid Function 2 (*N* = 3)	1200
	13	Hybrid Function 3 (*N* = 3)	1300
	14	Hybrid Function 4 (*N* = 4)	1400
	15	Hybrid Function 5 (*N* = 4)	1500
	16	Hybrid Function 6 (*N* = 4)	1600
	17	Hybrid Function 6 (*N* = 5)	1700
	18	Hybrid Function 6 (*N* = 5)	1800
	19	Hybrid Function 6 (*N* = 5)	1900
	20	Hybrid Function 6 (*N* = 6)	2000
Composition Functions	21	Composition Function 1 (*N* = 3)	2100
	22	Composition Function 2 (*N* = 3)	2200
	23	Composition Function 3 (*N* = 4)	2300
	24	Composition Function 4 (*N* = 4)	2400
	25	Composition Function 5 (*N* = 5)	2500
	26	Composition Function 6 (*N* = 5)	2600
	27	Composition Function 7 (*N* = 6)	2700
	28	Composition Function 8 (*N* = 6)	2800
	29	Composition Function 9 (*N* = 3)	2900
	30	Composition Function 10 (*N* = 3)	3000
Search Range: [−100,100]			

**Table 3:** Test results of the algorithm improved by different strategies.

		WOA	HHO	BOA	DBO	GJO	SWO	KOA	SABO	ZOA	MZOA
F1	min	3.74 × 10^−166^	5.31 × 10^−209^	1.57 × 10^−14^	5.65 × 10^−307^	6.38 × 10^−119^	1.18 × 10^−8^	4.56 × 10^+4^	0.00 × 10^+0^	0.00 × 10^+0^	0.00 × 10^+0^
F1	std	4.28 × 10^−150^	0.00 × 10^+0^	9.64 × 10^−16^	0.00 × 10^+0^	3.22 × 10^−111^	7.66 × 10^+0^	4.58 × 10^+3^	0.00 × 10^+0^	0.00 × 10^+0^	0.00 × 10^+0^
F1	avg	1.46 × 10^−150^	9.25 × 10^−187^	1.77 × 10^−14^	3.23 × 10^−240^	9.06 × 10^−112^	1.68 × 10^+0^	5.39 × 10^+4^	0.00 × 10^+0^	0.00 × 10^+0^	0.00 × 10^+0^
F2	min	1.12 × 10^−116^	1.39 × 10^−111^	1.52 × 10^−12^	1.98 × 10^−161^	7.41 × 10^−68^	3.49 × 10^−6^	2.35 × 10^+2^	1.36 × 10^−226^	1.82 × 10^−272^	0.00 × 10^+0^
F2	std	1.13 × 10^−103^	7.55 × 10^−94^	2.73 × 10^−12^	1.92 × 10^−120^	9.70 × 10^−66^	3.76 × 10^−1^	6.10 × 10^+9^	0.00 × 10^+0^	0.00 × 10^+0^	0.00 × 10^+0^
F2	avg	2.35 × 10^−104^	1.38 × 10^−94^	1.01 × 10^−11^	3.50 × 10^−121^	4.84 × 10^−66^	1.49 × 10^−1^	3.25 × 10^+9^	8.78 × 10^−224^	6.47 × 10^−266^	0.00 × 10^+0^
F3	min	7.09 × 10^+3^	2.75 × 10^−187^	1.58 × 10^−14^	2.47 × 10^−275^	4.97 × 10^−49^	2.45 × 10^−6^	5.82 × 10^+4^	3.31 × 10^−167^	0.00 × 10^+0^	0.00 × 10^+0^
F3	std	1.03 × 10^+4^	6.20 × 10^−130^	1.04 × 10^−15^	2.83 × 10^−115^	9.89 × 10^−37^	5.18 × 10^+1^	1.03 × 10^+4^	6.75 × 10^−38^	0.00 × 10^+0^	0.00 × 10^+0^
F3	avg	2.18 × 10^+4^	1.13 × 10^−130^	1.80 × 10^−14^	5.17 × 10^−116^	2.18 × 10^−37^	1.68 × 10^+1^	7.79 × 10^+4^	1.23 × 10^−38^	0.00 × 10^+0^	0.00 × 10^+0^
F4	min	5.29 × 10^−2^	1.20 × 10^−103^	1.03 × 10^−11^	1.71 × 10^−148^	3.62 × 10^−36^	6.49 × 10^−4^	7.44 × 10^+1^	6.22 × 10^−159^	6.76 × 10^−238^	0.00 × 10^+0^
F4	std	2.93 × 10^+1^	6.83 × 10^−92^	7.58 × 10^−13^	4.58 × 10^−99^	6.04 × 10^−32^	7.76 × 10^−1^	2.58 × 10^+0^	1.09 × 10^−155^	0.00 × 10^+0^	0.00 × 10^+0^
F4	avg	3.30 × 10^+1^	1.60 × 10^−92^	1.20 × 10^−11^	8.37 × 10^−100^	1.46 × 10^−32^	4.77 × 10^−1^	7.99 × 10^+1^	6.50 × 10^−156^	3.32 × 10^−231^	0.00 × 10^+0^
F5	min	2.62 × 10^+1^	1.34 × 10^−5^	2.88 × 10^+1^	2.46 × 10^+1^	2.62 × 10^+1^	2.89 × 10^+1^	8.14 × 10^+7^	2.70 × 10^+1^	2.68 × 10^+1^	2.28 × 10^+1^
F5	std	5.38 × 10^−1^	4.61 × 10^−3^	2.78 × 10^−2^	1.43 × 10^−1^	7.65 × 10^−1^	7.49 × 10^+2^	3.42 × 10^+7^	6.41 × 10^−1^	7.65 × 10^−1^	1.24 × 10^+0^
F5	avg	2.72 × 10^+1^	3.75 × 10^−3^	2.89 × 10^+1^	2.49 × 10^+1^	2.74 × 10^+1^	1.87 × 10^+2^	1.64 × 10^+8^	2.80 × 10^+1^	2.79 × 10^+1^	2.46 × 10^+1^
F6	min	1.21 × 10^−2^	1.49 × 10^−6^	3.47 × 10^+0^	5.69 × 10^−12^	1.75 × 10^+0^	2.99 × 10^+0^	3.83 × 10^+4^	1.11 × 10^+0^	1.21 × 10^+0^	8.84 × 10^−6^
F6	std	1.41 × 10^−1^	4.80 × 10^−5^	6.75 × 10^−1^	2.91 × 10^−7^	4.41 × 10^−1^	4.50 × 10^+1^	4.79 × 10^+3^	5.02 × 10^−1^	5.98 × 10^−1^	1.55 × 10^−1^
F6	avg	1.01 × 10^−1^	3.48 × 10^−5^	5.83 × 10^+0^	5.62 × 10^−8^	2.68 × 10^+0^	1.52 × 10^+1^	5.29 × 10^+4^	2.04 × 10^+0^	2.30 × 10^+0^	8.57 × 10^−2^
F7	min	1.24 × 10^−5^	4.70 × 10^−6^	2.11 × 10^−4^	3.51 × 10^−5^	2.79 × 10^−5^	1.55 × 10^−3^	5.83 × 10^+1^	4.56 × 10^−7^	1.04 × 10^−5^	4.08 × 10^−9^
F7	std	2.33 × 10^−3^	6.28 × 10^−5^	2.67 × 10^−4^	4.83 × 10^−4^	1.40 × 10^−4^	1.53 × 10^−2^	1.32 × 10^+1^	5.74 × 10^−5^	3.28 × 10^−5^	1.85 × 10^−5^
F7	avg	1.62 × 10^−3^	7.52 × 10^−5^	6.44 × 10^−4^	5.95 × 10^−4^	1.77 × 10^−4^	1.92 × 10^−2^	8.16 × 10^+1^	5.89 × 10^−5^	5.44 × 10^−5^	1.76 × 10^−5^
F8	min	−1.26 × 10^+4^	−1.26 × 10^+4^	−3.50 × 10^+3^	−1.25 × 10^+4^	−7.10 × 10^+3^	−6.09 × 10^+3^	−5.42 × 10^+3^	−4.16 × 10^+3^	−7.75 × 10^+3^	−1.08 × 10^+4^
F8	std	1.39 × 10^+3^	3.44 × 10^−1^	3.28 × 10^+2^	2.12 × 10^+3^	1.11 × 10^+3^	4.48 × 10^+2^	1.85 × 10^−12^	3.46 × 10^+2^	6.16 × 10^+2^	8.19 × 10^+2^
F8	avg	−1.15 × 10^+4^	−1.26 × 10^+4^	−3.96 × 10^+3^	−1.02 × 10^+4^	−4.24 × 10^+3^	−5.03 × 10^+3^	−5.42 × 10^+3^	−3.13 × 10^+3^	−6.79 × 10^+3^	−9.49 × 10^+3^
F9	min	0.00 × 10^+0^	0.00 × 10^+0^	0.00 × 10^+0^	0.00 × 10^+0^	0.00 × 10^+0^	3.39 × 10^−7^	3.38 × 10^+2^	0.00 × 10^+0^	0.00 × 10^+0^	0.00 × 10^+0^
F9	std	1.04 × 10^−14^	0.00 × 10^+0^	3.72 × 10^+1^	2.28 × 10^−9^	0.00 × 10^+0^	1.09 × 10^+0^	2.03 × 10^+1^	0.00 × 10^+0^	0.00 × 10^+0^	0.00 × 10^+0^
F9	avg	1.89 × 10^−15^	0.00 × 10^+0^	6.78 × 10^+0^	4.16 × 10^−10^	0.00 × 10^+0^	4.77 × 10^−1^	3.91 × 10^+2^	0.00 × 10^+0^	0.00 × 10^+0^	0.00 × 10^+0^
F10	min	8.88 × 10^−16^	8.88 × 10^−16^	5.66 × 10^−12^	8.88 × 10^−16^	4.44 × 10^−15^	1.83 × 10^−6^	2.00 × 10^+1^	4.44 × 10^−15^	8.88 × 10^−16^	8.88 × 10^−16^
F10	std	2.31 × 10^−15^	0.00 × 10^+0^	1.54 × 10^−12^	1.08 × 10^−15^	1.08 × 10^−15^	8.29 × 10^−1^	7.23 × 10^−15^	0.00 × 10^+0^	0.00 × 10^+0^	0.00 × 10^+0^
F10	avg	3.38 × 10^−15^	8.88 × 10^−16^	1.22 × 10^−11^	1.24 × 10^−15^	4.80 × 10^−15^	3.78 × 10^−1^	2.00 × 10^+1^	4.44 × 10^−15^	8.88 × 10^−16^	8.88 × 10^−16^
F11	min	0.00 × 10^+0^	0.00 × 10^+0^	0.00 × 10^+0^	0.00 × 10^+0^	0.00 × 10^+0^	2.97 × 10^−7^	4.35 × 10^+2^	0.00 × 10^+0^	0.00 × 10^+0^	0.00 × 10^+0^
F11	std	3.19 × 10^−2^	0.00 × 10^+0^	1.66 × 10^−15^	2.25 × 10^−3^	0.00 × 10^+0^	3.27 × 10^−1^	3.38 × 10^+1^	0.00 × 10^+0^	0.00 × 10^+0^	0.00 × 10^+0^
F11	avg	9.83 × 10^−3^	0.00 × 10^+0^	1.08 × 10^−15^	4.11 × 10^−4^	0.00 × 10^+0^	1.94 × 10^−1^	5.06 × 10^+2^	0.00 × 10^+0^	0.00 × 10^+0^	0.00 × 10^+0^
F12	min	1.24 × 10^−3^	2.03 × 10^−8^	3.02 × 10^−1^	7.66 × 10^−13^	9.70 × 10^−2^	1.31 × 10^−1^	1.52 × 10^+8^	6.66 × 10^−2^	4.38 × 10^−2^	2.78 × 10^−7^
F12	std	6.99 × 10^−3^	1.82 × 10^−6^	1.93 × 10^−1^	1.89 × 10^−2^	6.58 × 10^−2^	2.94 × 10^−1^	8.12 × 10^+7^	4.83 × 10^−2^	1.03 × 10^−1^	4.05 × 10^−3^
F12	avg	7.85 × 10^−3^	1.39 × 10^−6^	6.46 × 10^−1^	3.46 × 10^−3^	2.07 × 10^−1^	6.05 × 10^−1^	3.09 × 10^+8^	1.53 × 10^−1^	1.38 × 10^−1^	2.79 × 10^−3^
F13	min	1.05 × 10^−2^	2.58 × 10^−9^	2.28 × 10^+0^	7.93 × 10^−7^	1.35 × 10^+0^	2.04 × 10^+0^	4.73 × 10^+8^	6.71 × 10^−1^	1.59 × 10^+0^	4.09 × 10^−6^
F13	std	1.62 × 10^−1^	3.25 × 10^−5^	2.46 × 10^−1^	3.40 × 10^−1^	2.23 × 10^−1^	1.49 × 10^+0^	1.30 × 10^+8^	6.26 × 10^−1^	3.25 × 10^−1^	8.87 × 10^−2^
F13	avg	2.07 × 10^−1^	2.56 × 10^−5^	2.76 × 10^+0^	3.34 × 10^−1^	1.70 × 10^+0^	3.37 × 10^+0^	7.33 × 10^+8^	2.63 × 10^+0^	2.19 × 10^+0^	6.70 × 10^−2^
F14	min	9.98 × 10^−1^	9.98 × 10^−1^	9.98 × 10^−1^	9.98 × 10^−1^	9.98 × 10^−1^	9.98 × 10^−1^	9.98 × 10^−1^	9.98 × 10^−1^	9.98 × 10^−1^	9.98 × 10^−1^
F14	std	2.93 × 10^+0^	9.32 × 10^−1^	1.82 × 10^−1^	7.59 × 10^−1^	4.58 × 10^+0^	1.26 × 10^+0^	3.21 × 10^+0^	1.26 × 10^+0^	2.26 × 10^+0^	1.85 × 10^+0^
F14	avg	2.40 × 10^+0^	1.26 × 10^+0^	1.04 × 10^+0^	1.36 × 10^+0^	5.11 × 10^+0^	2.08 × 10^+0^	5.36 × 10^+0^	2.89 × 10^+0^	3.20 × 10^+0^	1.52 × 10^+0^
F15	min	3.09 × 10^−4^	3.08 × 10^−4^	3.09 × 10^−4^	3.07 × 10^−4^	3.07 × 10^−4^	4.20 × 10^−4^	2.06 × 10^−3^	3.33 × 10^−4^	3.07 × 10^−4^	3.07 × 10^−4^
F15	std	4.40 × 10^−4^	1.69 × 10^−4^	2.76 × 10^−5^	2.84 × 10^−4^	3.65 × 10^−3^	8.33 × 10^−4^	1.40 × 10^−2^	7.54 × 10^−4^	3.66 × 10^−3^	1.90 × 10^−5^
F15	avg	6.78 × 10^−4^	3.59 × 10^−4^	3.43 × 10^−4^	6.08 × 10^−4^	1.06 × 10^−3^	1.31 × 10^−3^	2.10 × 10^−2^	7.65 × 10^−4^	1.02 × 10^−3^	3.17 × 10^−4^
F16	min	−1.03 × 10^+0^	−1.03 × 10^+0^	−1.11 × 10^+0^	−1.03 × 10^+0^	−1.03 × 10^+0^	−1.03 × 10^+0^	−1.03 × 10^+0^	−1.03 × 10^+0^	−1.03 × 10^+0^	−1.03 × 10^+0^
F16	std	2.00 × 10^−10^	4.13 × 10^−11^	8.33 × 10^+3^	6.58 × 10^−16^	4.76 × 10^−7^	2.51 × 10^−4^	1.65 × 10^−1^	1.67 × 10^−2^	1.35 × 10^−10^	1.14 × 10^−11^
F16	avg	−1.03 × 10^+0^	−1.03 × 10^+0^	−1.16 × 10^+4^	−1.03 × 10^+0^	−1.03 × 10^+0^	−1.03 × 10^+0^	−8.83 × 10^−1^	−1.02 × 10^+0^	−1.03 × 10^+0^	−1.03 × 10^+0^
F17	min	3.98 × 10^−1^	3.98 × 10^−1^	3.98 × 10^−1^	3.98 × 10^−1^	3.98 × 10^−1^	3.98 × 10^−1^	4.02 × 10^−1^	3.98 × 10^−1^	3.98 × 10^−1^	3.98 × 10^−1^
F17	std	1.01 × 10^−6^	2.14 × 10^−6^	1.50 × 10^−3^	0.00 × 10^+0^	9.90 × 10^−6^	1.27 × 10^−2^	8.46 × 10^−2^	1.11 × 10^−1^	1.80 × 10^−8^	1.18 × 10^−9^
F17	avg	3.98 × 10^−1^	3.98 × 10^−1^	3.99 × 10^−1^	3.98 × 10^−1^	3.98 × 10^−1^	4.02 × 10^−1^	4.99 × 10^−1^	4.26 × 10^−1^	3.98 × 10^−1^	3.98 × 10^−1^
F18	min	3.00 × 10^+0^	3.00 × 10^+0^	3.00 × 10^+0^	3.00 × 10^+0^	3.00 × 10^+0^	3.00 × 10^+0^	3.15 × 10^+0^	3.00 × 10^+0^	3.00 × 10^+0^	3.00 × 10^+0^
F18	std	1.73 × 10^−5^	2.83 × 10^−8^	2.95 × 10^−1^	1.40 × 10^−15^	1.41 × 10^−6^	3.26 × 10^−2^	2.92 × 10^+0^	2.74 × 10^+0^	4.93 × 10^+0^	5.37 × 10^−8^
F18	avg	3.00 × 10^+0^	3.00 × 10^+0^	3.08 × 10^+0^	3.00 × 10^+0^	3.00 × 10^+0^	3.01 × 10^+0^	5.94 × 10^+0^	4.19 × 10^+0^	3.90 × 10^+0^	3.00 × 10^+0^
F19	min	−3.86 × 10^+0^	−3.86 × 10^+0^	−3.85 × 10^+0^	−3.86 × 10^+0^	−3.86 × 10^+0^	−3.86 × 10^+0^	−3.86 × 10^+0^	−3.86 × 10^+0^	−3.86 × 10^+0^	−3.86 × 10^+0^
F19	std	3.01 × 10^−3^	1.55 × 10^−3^	2.41 × 10^−1^	3.39 × 10^−3^	3.84 × 10^−3^	1.14 × 10^−3^	7.12 × 10^−2^	2.20 × 10^−1^	1.24 × 10^−4^	2.00 × 10^−3^
F19	avg	−3.86 × 10^+0^	−3.86 × 10^+0^	6.55 × 10^+4^	−3.86 × 10^+0^	−3.86 × 10^+0^	−3.86 × 10^+0^	−3.77 × 10^+0^	−3.63 × 10^+0^	−3.86 × 10^+0^	−3.86 × 10^+0^
F20	min	−3.32 × 10^+0^	−3.31 × 10^+0^	−2.44 × 10^+0^	−3.32 × 10^+0^	−3.32 × 10^+0^	−3.32 × 10^+0^	−3.17 × 10^+0^	−3.32 × 10^+0^	−3.32 × 10^+0^	−3.32 × 10^+0^
F20	std	8.46 × 10^−2^	8.06 × 10^−2^	1.55 × 10^−1^	1.40 × 10^−1^	1.28 × 10^−1^	6.02 × 10^−2^	2.73 × 10^−1^	7.58 × 10^−2^	3.67 × 10^−2^	9.65 × 10^−2^
F20	avg	−3.23 × 10^+0^	−3.17 × 10^+0^	6.55 × 10^+4^	−3.22 × 10^+0^	−3.10 × 10^+0^	−3.27 × 10^+0^	−2.64 × 10^+0^	−3.27 × 10^+0^	−3.31 × 10^+0^	−3.28 × 10^+0^
F21	min	−1.02 × 10^+1^	−1.01 × 10^+1^	−5.18 × 10^+0^	−1.02 × 10^+1^	−1.02 × 10^+1^	−1.01 × 10^+1^	−4.48 × 10^+0^	−5.05 × 10^+0^	−1.02 × 10^+1^	−1.02 × 10^+1^
F21	std	2.27 × 10^+0^	1.27 × 10^+0^	1.93 × 10^−1^	2.53 × 10^+0^	2.58 × 10^+0^	2.73 × 10^+0^	8.40 × 10^−1^	1.01 × 10^−1^	2.66 × 10^−4^	2.68 × 10^+0^
F21	avg	−9.05 × 10^+0^	−5.39 × 10^+0^	−4.73 × 10^+0^	−7.11 × 10^+0^	−8.37 × 10^+0^	−5.84 × 10^+0^	−1.43 × 10^+0^	−5.03 × 10^+0^	−1.02 × 10^+1^	−8.99 × 10^+0^
F22	min	−1.04 × 10^+1^	−1.04 × 10^+1^	−5.75 × 10^+0^	−1.04 × 10^+1^	−1.04 × 10^+1^	−1.01 × 10^+1^	−2.39 × 10^+0^	−9.98 × 10^+0^	−1.04 × 10^+1^	−1.04 × 10^+1^
F22	std	3.32 × 10^+0^	1.31 × 10^+0^	3.72 × 10^−1^	2.62 × 10^+0^	1.87 × 10^−3^	2.41 × 10^+0^	3.97 × 10^−1^	1.11 × 10^+0^	1.62 × 10^+0^	2.78 × 10^+0^
F22	avg	−7.46 × 10^+0^	−5.43 × 10^+0^	−4.68 × 10^+0^	−8.74 × 10^+0^	−1.04 × 10^+1^	−6.90 × 10^+0^	−1.44 × 10^+0^	−5.25 × 10^+0^	−9.87 × 10^+0^	−9.05 × 10^+0^
F23	min	−1.05 × 10^+1^	−1.04 × 10^+1^	−6.84 × 10^+0^	−1.05 × 10^+1^	−1.05 × 10^+1^	−1.04 × 10^+1^	−4.36 × 10^+0^	−1.00 × 10^+1^	−1.05 × 10^+1^	−1.05 × 10^+1^
F23	std	2.94 × 10^+0^	9.61 × 10^−1^	5.68 × 10^−1^	2.69 × 10^+0^	1.96 × 10^+0^	3.02 × 10^+0^	7.10 × 10^−1^	1.73 × 10^+0^	1.65 × 10^+0^	3.31 × 10^+0^
F23	avg	−8.37 × 10^+0^	−5.30 × 10^+0^	−4.64 × 10^+0^	−8.83 × 10^+0^	−9.91 × 10^+0^	−6.25 × 10^+0^	−1.80 × 10^+0^	−5.70 × 10^+0^	−1.00 × 10^+1^	−8.43 × 10^+0^

**Table 4:** Test results of the algorithms improved by different strategies.

		WOA	HHO	BOA	DBO	GJO	SWO	KOA	SABO	ZOA	MZOA
F1	min	5.27 × 10^+8^	1.99 × 10^+7^	3.22 × 10^+10^	1.23 × 10^+6^	3.83 × 10^+9^	8.86 × 10^+9^	5.38 × 10^+10^	3.37 × 10^+9^	2.96 × 10^+9^	6.76 × 10^+4^
F1	std	7.37 × 10^+8^	7.14 × 10^+6^	9.84 × 10^+9^	3.57 × 10^+7^	4.54 × 10^+9^	5.19 × 10^+9^	1.48 × 10^+10^	3.28 × 10^+9^	4.07 × 10^+9^	1.06 × 10^+7^
F1	avg	1.44 × 10^+9^	3.32 × 10^+7^	5.27 × 10^+10^	5.47 × 10^+7^	1.15 × 10^+10^	1.73 × 10^+10^	9.08 × 10^+10^	8.34 × 10^+9^	1.04 × 10^+10^	4.35 × 10^+6^
F3	min	1.05 × 10^+5^	3.32 × 10^+4^	8.03 × 10^+4^	4.67 × 10^+4^	3.14 × 10^+4^	6.51 × 10^+4^	1.46 × 10^+5^	4.59 × 10^+4^	2.22 × 10^+4^	1.67 × 10^+4^
F3	std	6.62 × 10^+4^	7.79 × 10^+3^	1.59 × 10^+4^	1.77 × 10^+4^	1.45 × 10^+4^	2.01 × 10^+4^	1.13 × 10^+6^	1.38 × 10^+4^	1.00 × 10^+4^	2.41 × 10^+4^
F3	avg	2.61 × 10^+5^	4.86 × 10^+4^	1.02 × 10^+5^	8.83 × 10^+4^	5.41 × 10^+4^	1.01 × 10^+5^	4.88 × 10^+5^	7.45 × 10^+4^	4.15 × 10^+4^	6.31 × 10^+4^
F4	min	6.41 × 10^+2^	4.76 × 10^+2^	1.35 × 10^+4^	4.96 × 10^+2^	7.45 × 10^+2^	2.51 × 10^+3^	1.26 × 10^+4^	8.25 × 10^+2^	5.82 × 10^+2^	4.12 × 10^+2^
F4	std	1.36 × 10^+2^	3.04 × 10^+1^	5.49 × 10^+3^	7.29 × 10^+1^	1.24 × 10^+3^	1.08 × 10^+3^	7.07 × 10^+3^	1.65 × 10^+3^	1.56 × 10^+3^	3.52 × 10^+1^
F4	avg	8.25 × 10^+2^	5.53 × 10^+2^	2.43 × 10^+4^	6.19 × 10^+2^	2.03 × 10^+3^	3.84 × 10^+3^	2.86 × 10^+4^	2.26 × 10^+3^	1.99 × 10^+3^	4.90 × 10^+2^
F5	min	7.21 × 10^+2^	6.89 × 10^+2^	8.67 × 10^+2^	6.50 × 10^+2^	6.38 × 10^+2^	7.70 × 10^+2^	9.84 × 10^+2^	7.73 × 10^+2^	6.63 × 10^+2^	5.75 × 10^+2^
F5	std	6.53 × 10^+1^	2.40 × 10^+1^	1.98 × 10^+1^	6.01 × 10^+1^	5.27 × 10^+1^	3.44 × 10^+1^	4.22 × 10^+1^	3.81 × 10^+1^	2.75 × 10^+1^	3.77 × 10^+1^
F5	avg	8.27 × 10^+2^	7.37 × 10^+2^	9.02 × 10^+2^	7.44 × 10^+2^	7.08 × 10^+2^	8.34 × 10^+2^	1.08 × 10^+3^	8.30 × 10^+2^	7.09 × 10^+2^	6.64 × 10^+2^
F6	min	6.45 × 10^+2^	6.51 × 10^+2^	6.71 × 10^+2^	6.22 × 10^+2^	6.24 × 10^+2^	6.48 × 10^+2^	6.93 × 10^+2^	6.33 × 10^+2^	6.41 × 10^+2^	6.12 × 10^+2^
F6	std	1.20 × 10^+1^	6.07 × 10^+0^	6.97 × 10^+0^	1.39 × 10^+1^	8.30 × 10^+0^	8.32 × 10^+0^	9.57 × 10^+0^	1.18 × 10^+1^	4.64 × 10^+0^	9.39 × 10^+0^
F6	avg	6.74 × 10^+2^	6.64 × 10^+2^	6.83 × 10^+2^	6.49 × 10^+2^	6.39 × 10^+2^	6.63 × 10^+2^	7.17 × 10^+2^	6.58 × 10^+2^	6.50 × 10^+2^	6.30 × 10^+2^
F7	min	1.25 × 10^+3^	1.31 × 10^+3^	1.50 × 10^+3^	8.85 × 10^+2^	8.79 × 10^+2^	1.20 × 10^+3^	2.50 × 10^+3^	1.06 × 10^+3^	1.07 × 10^+3^	8.69 × 10^+2^
F7	std	1.01 × 10^+2^	7.59 × 10^+1^	4.23 × 10^+1^	1.03 × 10^+2^	8.29 × 10^+1^	9.65 × 10^+1^	2.33 × 10^+2^	8.29 × 10^+1^	9.08 × 10^+1^	9.13 × 10^+1^
F7	avg	1.46 × 10^+3^	1.45 × 10^+3^	1.62 × 10^+3^	1.04 × 10^+3^	1.07 × 10^+3^	1.36 × 10^+3^	2.99 × 10^+3^	1.22 × 10^+3^	1.29 × 10^+3^	9.98 × 10^+2^
F8	min	1.04 × 10^+3^	9.72 × 10^+2^	1.17 × 10^+3^	9.38 × 10^+2^	9.57 × 10^+2^	1.06 × 10^+3^	1.28 × 10^+3^	1.03 × 10^+3^	9.27 × 10^+2^	8.82 × 10^+2^
F8	std	4.60 × 10^+1^	3.61 × 10^+1^	2.34 × 10^+1^	5.02 × 10^+1^	4.05 × 10^+1^	3.01 × 10^+1^	3.91 × 10^+1^	3.14 × 10^+1^	3.02 × 10^+1^	3.51 × 10^+1^
F8	avg	1.11 × 10^+3^	1.04 × 10^+3^	1.21 × 10^+3^	1.06 × 10^+3^	1.01 × 10^+3^	1.13 × 10^+3^	1.37 × 10^+3^	1.09 × 10^+3^	1.01 × 10^+3^	9.46 × 10^+2^
F9	min	6.12 × 10^+3^	7.86 × 10^+3^	1.04 × 10^+4^	3.39 × 10^+3^	3.12 × 10^+3^	6.08 × 10^+3^	2.36 × 10^+4^	3.87 × 10^+3^	3.13 × 10^+3^	2.56 × 10^+3^
F9	std	3.61 × 10^+3^	1.12 × 10^+3^	1.52 × 10^+3^	2.47 × 10^+3^	2.56 × 10^+3^	2.84 × 10^+3^	3.93 × 10^+3^	3.03 × 10^+3^	9.40 × 10^+2^	1.26 × 10^+3^
F9	avg	1.23 × 10^+4^	1.09 × 10^+4^	1.46 × 10^+4^	7.93 × 10^+3^	7.42 × 10^+3^	1.30 × 10^+4^	3.17 × 10^+4^	8.49 × 10^+3^	5.67 × 10^+3^	5.62 × 10^+3^
F10	min	4.82 × 10^+3^	3.99 × 10^+3^	8.02 × 10^+3^	4.09 × 10^+3^	4.39 × 10^+3^	7.36 × 10^+3^	8.72 × 10^+3^	7.90 × 10^+3^	3.93 × 10^+3^	2.90 × 10^+3^
F10	std	9.19 × 10^+2^	7.14 × 10^+2^	3.60 × 10^+2^	1.15 × 10^+3^	1.77 × 10^+3^	5.02 × 10^+2^	4.51 × 10^+2^	3.61 × 10^+2^	3.92 × 10^+2^	8.64 × 10^+2^
F10	avg	6.83 × 10^+3^	5.62 × 10^+3^	8.90 × 10^+3^	6.25 × 10^+3^	6.81 × 10^+3^	8.56 × 10^+3^	9.73 × 10^+3^	8.75 × 10^+3^	4.85 × 10^+3^	5.46 × 10^+3^
F11	min	2.25 × 10^+3^	1.19 × 10^+3^	3.33 × 10^+3^	1.35 × 10^+3^	1.69 × 10^+3^	2.25 × 10^+3^	8.65 × 10^+3^	2.61 × 10^+3^	1.35 × 10^+3^	1.14 × 10^+3^
F11	std	2.44 × 10^+3^	4.76 × 10^+1^	2.19 × 10^+3^	6.81 × 10^+2^	2.01 × 10^+3^	1.35 × 10^+3^	6.52 × 10^+3^	2.06 × 10^+3^	8.15 × 10^+2^	9.46 × 10^+1^
F11	avg	6.68 × 10^+3^	1.30 × 10^+3^	7.58 × 10^+3^	1.72 × 10^+3^	3.89 × 10^+3^	4.64 × 10^+3^	2.35 × 10^+4^	4.80 × 10^+3^	2.21 × 10^+3^	1.30 × 10^+3^
F12	min	4.99 × 10^+7^	7.25 × 10^+6^	6.10 × 10^+9^	1.06 × 10^+6^	4.82 × 10^+7^	6.13 × 10^+8^	9.49 × 10^+9^	6.09 × 10^+7^	8.15 × 10^+6^	4.50 × 10^+5^
F12	std	1.77 × 10^+8^	2.24 × 10^+7^	3.74 × 10^+9^	5.15 × 10^+7^	3.05 × 10^+8^	9.16 × 10^+8^	3.49 × 10^+9^	7.63 × 10^+8^	1.25 × 10^+9^	3.70 × 10^+6^
F12	avg	2.74 × 10^+8^	2.85 × 10^+7^	1.35 × 10^+10^	3.15 × 10^+7^	6.17 × 10^+8^	1.66 × 10^+9^	1.76 × 10^+10^	5.43 × 10^+8^	6.80 × 10^+8^	4.86 × 10^+6^
F13	min	9.33 × 10^+4^	2.24 × 10^+5^	4.11 × 10^+9^	3.75 × 10^+4^	1.46 × 10^+5^	7.56 × 10^+7^	5.93 × 10^+9^	1.43 × 10^+6^	2.13 × 10^+4^	9.04 × 10^+4^
F13	std	5.18 × 10^+6^	3.29 × 10^+5^	5.01 × 10^+9^	4.70 × 10^+6^	2.18 × 10^+8^	5.43 × 10^+8^	4.50 × 10^+9^	4.30 × 10^+7^	5.97 × 10^+8^	1.08 × 10^+5^
F13	avg	3.07 × 10^+6^	6.61 × 10^+5^	1.14 × 10^+10^	2.95 × 10^+6^	2.15 × 10^+8^	5.91 × 10^+8^	1.41 × 10^+10^	2.71 × 10^+7^	1.49 × 10^+8^	2.69 × 10^+5^
F14	min	1.87 × 10^+4^	2.40 × 10^+4^	9.93 × 10^+4^	5.08 × 10^+3^	2.34 × 10^+4^	9.22 × 10^+4^	8.79 × 10^+4^	9.08 × 10^+4^	5.43 × 10^+3^	3.43 × 10^+3^
F14	std	3.51 × 10^+6^	1.01 × 10^+6^	4.41 × 10^+6^	4.36 × 10^+5^	7.21 × 10^+5^	7.79 × 10^+5^	6.96 × 10^+6^	6.78 × 10^+5^	7.61 × 10^+5^	7.99 × 10^+4^
F14	avg	3.05 × 10^+6^	1.02 × 10^+6^	3.75 × 10^+6^	2.92 × 10^+5^	7.07 × 10^+5^	9.05 × 10^+5^	1.28 × 10^+7^	7.80 × 10^+5^	8.23 × 10^+5^	8.56 × 10^+4^
F15	min	5.85 × 10^+4^	3.09 × 10^+4^	3.65 × 10^+7^	5.11 × 10^+3^	5.11 × 10^+4^	1.17 × 10^+6^	5.78 × 10^+8^	5.89 × 10^+4^	2.30 × 10^+4^	3.70 × 10^+3^
F15	std	1.59 × 10^+6^	5.81 × 10^+4^	5.20 × 10^+8^	1.28 × 10^+5^	1.57 × 10^+7^	1.75 × 10^+7^	9.96 × 10^+8^	3.29 × 10^+5^	1.78 × 10^+6^	2.03 × 10^+4^
F15	avg	1.33 × 10^+6^	1.11 × 10^+5^	5.74 × 10^+8^	9.67 × 10^+4^	6.57 × 10^+6^	1.93 × 10^+7^	2.34 × 10^+9^	4.01 × 10^+5^	1.23 × 10^+6^	2.11 × 10^+4^
F16	min	3.13 × 10^+3^	2.41 × 10^+3^	4.80 × 10^+3^	2.41 × 10^+3^	2.02 × 10^+3^	3.53 × 10^+3^	5.54 × 10^+3^	3.57 × 10^+3^	2.63 × 10^+3^	2.14 × 10^+3^
F16	std	5.83 × 10^+2^	4.64 × 10^+2^	2.42 × 10^+3^	3.81 × 10^+2^	4.63 × 10^+2^	3.47 × 10^+2^	6.90 × 10^+2^	3.60 × 10^+2^	3.06 × 10^+2^	3.23 × 10^+2^
F16	avg	4.08 × 10^+3^	3.45 × 10^+3^	8.12 × 10^+3^	3.31 × 10^+3^	3.10 × 10^+3^	4.22 × 10^+3^	6.65 × 10^+3^	4.09 × 10^+3^	3.05 × 10^+3^	2.77 × 10^+3^
F17	min	2.25 × 10^+3^	1.86 × 10^+3^	3.07 × 10^+3^	2.12 × 10^+3^	1.84 × 10^+3^	1.97 × 10^+3^	3.34 × 10^+3^	2.41 × 10^+3^	1.97 × 10^+3^	1.85 × 10^+3^
F17	std	2.77 × 10^+2^	3.32 × 10^+2^	6.76 × 10^+3^	2.23 × 10^+2^	2.50 × 10^+2^	2.97 × 10^+2^	1.82 × 10^+3^	2.42 × 10^+2^	3.07 × 10^+2^	2.01 × 10^+2^
F17	avg	2.74 × 10^+3^	2.54 × 10^+3^	8.92 × 10^+3^	2.54 × 10^+3^	2.26 × 10^+3^	2.77 × 10^+3^	5.32 × 10^+3^	2.89 × 10^+3^	2.43 × 10^+3^	2.34 × 10^+3^
F18	min	3.32 × 10^+5^	1.36 × 10^+5^	7.54 × 10^+5^	1.12 × 10^+5^	2.37 × 10^+5^	2.51 × 10^+5^	2.44 × 10^+7^	1.19 × 10^+5^	8.18 × 10^+4^	9.64 × 10^+4^
F18	std	1.22 × 10^+7^	4.27 × 10^+6^	7.39 × 10^+7^	6.94 × 10^+6^	3.21 × 10^+6^	5.62 × 10^+6^	1.30 × 10^+8^	7.52 × 10^+6^	2.54 × 10^+6^	1.25 × 10^+6^
F18	avg	1.05 × 10^+7^	2.50 × 10^+6^	5.65 × 10^+7^	4.76 × 10^+6^	2.24 × 10^+6^	4.88 × 10^+6^	1.57 × 10^+8^	3.74 × 10^+6^	2.30 × 10^+6^	9.57 × 10^+5^
F19	min	3.31 × 10^+5^	2.62 × 10^+5^	1.11 × 10^+8^	7.50 × 10^+3^	4.76 × 10^+4^	6.29 × 10^+5^	9.15 × 10^+8^	1.36 × 10^+5^	6.66 × 10^+4^	2.23 × 10^+3^
F19	std	1.23 × 10^+7^	4.43 × 10^+5^	6.45 × 10^+8^	7.51 × 10^+6^	7.56 × 10^+6^	3.61 × 10^+7^	1.17 × 10^+9^	3.38 × 10^+6^	3.08 × 10^+7^	1.74 × 10^+4^
F19	avg	1.44 × 10^+7^	7.04 × 10^+5^	7.19 × 10^+8^	3.65 × 10^+6^	3.69 × 10^+6^	3.45 × 10^+7^	3.32 × 10^+9^	3.94 × 10^+6^	1.10 × 10^+7^	1.71 × 10^+4^
F20	min	2.42 × 10^+3^	2.33 × 10^+3^	2.71 × 10^+3^	2.34 × 10^+3^	2.23 × 10^+3^	2.55 × 10^+3^	3.09 × 10^+3^	2.70 × 10^+3^	2.24 × 10^+3^	2.28 × 10^+3^
F20	std	2.12 × 10^+2^	2.04 × 10^+2^	1.59 × 10^+2^	1.89 × 10^+2^	1.58 × 10^+2^	1.98 × 10^+2^	1.74 × 10^+2^	1.60 × 10^+2^	1.13 × 10^+2^	1.52 × 10^+2^
F20	avg	2.90 × 10^+3^	2.83 × 10^+3^	3.04 × 10^+3^	2.72 × 10^+3^	2.56 × 10^+3^	3.00 × 10^+3^	3.46 × 10^+3^	3.06 × 10^+3^	2.44 × 10^+3^	2.54 × 10^+3^
F21	min	2.49 × 10^+3^	2.46 × 10^+3^	2.41 × 10^+3^	2.44 × 10^+3^	2.43 × 10^+3^	2.53 × 10^+3^	2.77 × 10^+3^	2.53 × 10^+3^	2.45 × 10^+3^	2.38 × 10^+3^
F21	std	6.86 × 10^+1^	5.35 × 10^+1^	1.08 × 10^+2^	5.60 × 10^+1^	2.91 × 10^+1^	3.23 × 10^+1^	2.62 × 10^+1^	3.32 × 10^+1^	3.25 × 10^+1^	3.11 × 10^+1^
F21	avg	2.62 × 10^+3^	2.58 × 10^+3^	2.69 × 10^+3^	2.54 × 10^+3^	2.48 × 10^+3^	2.61 × 10^+3^	2.83 × 10^+3^	2.59 × 10^+3^	2.50 × 10^+3^	2.44 × 10^+3^
F22	min	2.58 × 10^+3^	2.33 × 10^+3^	4.50 × 10^+3^	2.32 × 10^+3^	2.86 × 10^+3^	3.83 × 10^+3^	9.87 × 10^+3^	3.25 × 10^+3^	2.94 × 10^+3^	2.30 × 10^+3^
F22	std	1.98 × 10^+3^	1.83 × 10^+3^	1.48 × 10^+3^	2.43 × 10^+3^	1.99 × 10^+3^	1.77 × 10^+3^	4.65 × 10^+2^	7.45 × 10^+2^	1.10 × 10^+3^	1.52 × 10^+3^
F22	avg	7.20 × 10^+3^	6.71 × 10^+3^	7.23 × 10^+3^	4.85 × 10^+3^	4.85 × 10^+3^	5.73 × 10^+3^	1.12 × 10^+4^	4.12 × 10^+3^	5.39 × 10^+3^	2.88 × 10^+3^
F23	min	2.93 × 10^+3^	3.00 × 10^+3^	3.28 × 10^+3^	2.86 × 10^+3^	2.79 × 10^+3^	3.01 × 10^+3^	3.41 × 10^+3^	3.00 × 10^+3^	2.99 × 10^+3^	2.73 × 10^+3^
F23	std	8.03 × 10^+1^	1.58 × 10^+2^	1.47 × 10^+2^	8.19 × 10^+1^	5.53 × 10^+1^	7.24 × 10^+1^	1.33 × 10^+2^	1.14 × 10^+2^	1.14 × 10^+2^	4.20 × 10^+1^
F23	avg	3.09 × 10^+3^	3.24 × 10^+3^	3.50 × 10^+3^	2.99 × 10^+3^	2.91 × 10^+3^	3.12 × 10^+3^	3.64 × 10^+3^	3.17 × 10^+3^	3.20 × 10^+3^	2.80 × 10^+3^
F24	min	3.04 × 10^+3^	3.23 × 10^+3^	3.63 × 10^+3^	3.01 × 10^+3^	2.96 × 10^+3^	3.21 × 10^+3^	3.69 × 10^+3^	3.11 × 10^+3^	3.33 × 10^+3^	2.89 × 10^+3^
F24	std	9.67 × 10^+1^	1.34 × 10^+2^	2.30 × 10^+2^	8.88 × 10^+1^	6.52 × 10^+1^	5.22 × 10^+1^	1.08 × 10^+2^	1.11 × 10^+2^	9.19 × 10^+1^	3.64 × 10^+1^
F24	avg	3.22 × 10^+3^	3.43 × 10^+3^	3.97 × 10^+3^	3.18 × 10^+3^	3.09 × 10^+3^	3.29 × 10^+3^	3.97 × 10^+3^	3.27 × 10^+3^	3.49 × 10^+3^	2.98 × 10^+3^
F25	min	3.03 × 10^+3^	2.90 × 10^+3^	4.83 × 10^+3^	2.89 × 10^+3^	3.00 × 10^+3^	3.35 × 10^+3^	8.30 × 10^+3^	3.08 × 10^+3^	3.01 × 10^+3^	2.88 × 10^+3^
F25	std	5.65 × 10^+1^	2.70 × 10^+1^	5.60 × 10^+2^	5.97 × 10^+1^	1.21 × 10^+2^	3.01 × 10^+2^	2.01 × 10^+3^	1.54 × 10^+2^	1.83 × 10^+2^	1.52 × 10^+1^
F25	avg	3.12 × 10^+3^	2.95 × 10^+3^	5.94 × 10^+3^	2.97 × 10^+3^	3.21 × 10^+3^	3.79 × 10^+3^	1.28 × 10^+4^	3.26 × 10^+3^	3.21 × 10^+3^	2.90 × 10^+3^
F26	min	4.83 × 10^+3^	3.46 × 10^+3^	9.42 × 10^+3^	3.52 × 10^+3^	5.31 × 10^+3^	5.95 × 10^+3^	1.12 × 10^+4^	5.50 × 10^+3^	5.79 × 10^+3^	2.81 × 10^+3^
F26	std	1.30 × 10^+3^	1.24 × 10^+3^	8.30 × 10^+2^	1.17 × 10^+3^	6.09 × 10^+2^	6.95 × 10^+2^	1.01 × 10^+3^	7.46 × 10^+2^	6.97 × 10^+2^	1.39 × 10^+3^
F26	avg	8.20 × 10^+3^	7.73 × 10^+3^	1.17 × 10^+4^	6.51 × 10^+3^	6.09 × 10^+3^	8.09 × 10^+3^	1.35 × 10^+4^	8.24 × 10^+3^	8.34 × 10^+3^	4.83 × 10^+3^
F27	min	3.28 × 10^+3^	3.26 × 10^+3^	3.53 × 10^+3^	3.26 × 10^+3^	3.27 × 10^+3^	3.48 × 10^+3^	4.05 × 10^+3^	3.27 × 10^+3^	3.56 × 10^+3^	3.20 × 10^+3^
F27	std	8.97 × 10^+1^	1.94 × 10^+2^	3.34 × 10^+2^	5.26 × 10^+1^	7.58 × 10^+1^	9.58 × 10^+1^	2.72 × 10^+2^	1.06 × 10^+2^	2.38 × 10^+2^	1.75 × 10^+1^
F27	avg	3.44 × 10^+3^	3.48 × 10^+3^	4.22 × 10^+3^	3.33 × 10^+3^	3.35 × 10^+3^	3.66 × 10^+3^	4.69 × 10^+3^	3.45 × 10^+3^	3.99 × 10^+3^	3.24 × 10^+3^
F28	min	3.39 × 10^+3^	3.29 × 10^+3^	6.93 × 10^+3^	3.25 × 10^+3^	3.56 × 10^+3^	3.77 × 10^+3^	7.61 × 10^+3^	3.48 × 10^+3^	3.46 × 10^+3^	3.21 × 10^+3^
F28	std	6.06 × 10^+2^	3.05 × 10^+1^	5.89 × 10^+2^	4.99 × 10^+2^	3.34 × 10^+2^	4.29 × 10^+2^	9.61 × 10^+2^	4.48 × 10^+2^	3.84 × 10^+2^	3.57 × 10^+1^
F28	avg	3.69 × 10^+3^	3.33 × 10^+3^	8.20 × 10^+3^	3.49 × 10^+3^	3.95 × 10^+3^	4.66 × 10^+3^	9.89 × 10^+3^	4.10 × 10^+3^	3.96 × 10^+3^	3.26 × 10^+3^
F29	min	4.16 × 10^+3^	4.01 × 10^+3^	5.93 × 10^+3^	3.76 × 10^+3^	3.86 × 10^+3^	4.43 × 10^+3^	6.46 × 10^+3^	4.53 × 10^+3^	4.02 × 10^+3^	3.62 × 10^+3^
F29	std	5.93 × 10^+2^	3.65 × 10^+2^	7.45 × 10^+3^	3.46 × 10^+2^	3.07 × 10^+2^	4.08 × 10^+2^	2.47 × 10^+3^	4.85 × 10^+2^	5.27 × 10^+2^	2.31 × 10^+2^
F29	avg	5.34 × 10^+3^	4.65 × 10^+3^	1.35 × 10^+4^	4.47 × 10^+3^	4.23 × 10^+3^	5.20 × 10^+3^	9.70 × 10^+3^	5.74 × 10^+3^	5.09 × 10^+3^	4.04 × 10^+3^
F30	min	2.32 × 10^+6^	1.34 × 10^+6^	1.01 × 10^+8^	3.96 × 10^+4^	5.10 × 10^+6^	1.04 × 10^+7^	1.35 × 10^+9^	3.92 × 10^+6^	1.95 × 10^+6^	2.13 × 10^+4^
F30	std	4.16 × 10^+7^	3.83 × 10^+6^	1.22 × 10^+9^	6.07 × 10^+6^	3.43 × 10^+7^	3.29 × 10^+7^	4.80 × 10^+8^	2.02 × 10^+7^	2.29 × 10^+7^	1.48 × 10^+5^
F30	avg	4.51 × 10^+7^	6.05 × 10^+6^	1.61 × 10^+9^	4.64 × 10^+6^	4.08 × 10^+7^	4.46 × 10^+7^	2.00 × 10^+9^	3.03 × 10^+7^	2.30 × 10^+7^	1.53 × 10^+5^

**Table 5:** Extension/compression spring optimization problem solving results.

Algorithms	*d*	*D*	*P*	Best	std	Mean
MZOA	5.00 × 10^−2^	7.70 × 10^−1^	9.84 × 10^−1^	1.15 × 10^−1^	3.68 × 10^−4^	1.15 × 10^−1^
ZOA	5.00 × 10^−2^	6.08 × 10^−1^	2.00 × 10^+0^	1.22 × 10^−1^	1.53 × 10^−3^	1.22 × 10^−1^
WOA	5.00 × 10^−2^	6.08 × 10^−1^	2.00 × 10^+0^	1.22 × 10^−1^	4.32 × 10^−2^	1.47 × 10^−1^
HHO	5.00 × 10^−2^	6.08 × 10^−1^	2.00 × 10^+0^	1.22 × 10^−1^	7.23 × 10^−3^	1.24 × 10^−1^
BOA	5.00 × 10^−2^	8.64 × 10^−1^	7.10 × 10^−1^	1.17 × 10^−1^	2.15 × 10^+2^	1.29 × 10^+2^
DBO	5.00 × 10^−2^	6.08 × 10^−1^	2.00 × 10^+0^	1.22 × 10^−1^	4.70 × 10^−16^	1.22 × 10^−1^
GJO	5.00 × 10^−2^	6.08 × 10^−1^	2.00 × 10^+0^	1.22 × 10^−1^	1.95 × 10^−4^	1.22 × 10^−1^
SWO	5.52 × 10^−2^	7.61 × 10^−1^	3.23 × 10^+0^	2.20 × 10^−1^	3.62 × 10^+2^	2.99 × 10^+2^
KOA	5.52 × 10^−2^	1.25 × 10^+0^	2.35 × 10^+0^	3.00 × 10^−1^	3.64 × 10^+2^	1.75 × 10^+2^
SABO	5.00 × 10^−2^	6.07 × 10^−1^	2.00 × 10^+0^	1.22 × 10^−1^	9.45 × 10^−3^	1.37 × 10^−1^

**Table 6:** Cantilever beam design problem solving results.

Algorithms	*x_1_*	*x_2_*	*x_3_*	*x_4_*	*x_5_*	Best	std	Mean
MZOA	5.95 × 10^+0^	5.31 × 10^+0^	4.48 × 10^+0^	3.53 × 10^+0^	2.19 × 10^+0^	1.34 × 10^+1^	1.55 × 10^−2^	1.34 × 10^+1^
ZOA	5.99 × 10^+0^	5.30 × 10^+0^	4.48 × 10^+0^	3.50 × 10^+0^	2.20 × 10^+0^	1.34 × 10^+1^	1.50 × 10^+0^	1.43 × 10^+1^
WOA	6.11 × 10^+0^	1.03 × 10^+1^	4.53 × 10^+0^	8.52 × 10^+0^	1.27 × 10^+0^	1.91 × 10^+1^	1.16 × 10^+1^	3.02 × 10^+1^
HHO	5.94 × 10^+0^	5.10 × 10^+0^	4.49 × 10^+0^	3.82 × 10^+0^	2.18 × 10^+0^	1.34 × 10^+1^	3.35 × 10^−1^	1.37 × 10^+1^
BOA	5.93 × 10^+0^	5.28 × 10^+0^	4.97 × 10^+0^	3.64 × 10^+0^	2.52 × 10^+0^	1.39 × 10^+1^	7.55 × 10^−1^	1.52 × 10^+1^
DBO	5.95 × 10^+0^	5.32 × 10^+0^	4.60 × 10^+0^	3.41 × 10^+0^	2.20 × 10^+0^	1.34 × 10^+1^	4.30 × 10^−2^	1.34 × 10^+1^
GJO	6.09 × 10^+0^	5.34 × 10^+0^	4.46 × 10^+0^	3.44 × 10^+0^	2.16 × 10^+0^	1.34 × 10^+1^	2.53 × 10^−2^	1.34 × 10^+1^
SWO	2.38 × 10^+1^	1.34 × 10^+1^	1.49 × 10^+1^	1.59 × 10^+1^	5.80 × 10^+0^	4.60 × 10^+1^	1.88 × 10^+1^	7.88 × 10^+1^
KOA	2.75 × 10^+1^	1.38 × 10^+1^	4.48 × 10^+0^	3.91 × 10^+1^	1.61 × 10^+1^	6.28 × 10^+1^	1.08 × 10^+1^	7.76 × 10^+1^
SABO	8.31 × 10^+0^	6.15 × 10^+0^	3.39 × 10^+0^	3.11 × 10^+0^	2.90 × 10^+0^	1.56 × 10^+1^	2.01 × 10^+0^	1.73 × 10^+1^

**Table 7:** Performance comparison of algorithms for maps of different sizes.

		MZOA	ZOA	WOA	HHO	BOA	DBO	GJO	SWO	KOA	SABO
20 × 20	min	28.130	28.583	28.583	28.337	28.278	28.509	28.204	28.583	31.594	28.382
	std	1.385	0.659	1.195	0.666	0.732	0.812	1.056	1.764	1.465	1.266
	avg	29.369	29.595	29.953	29.616	29.742	29.593	29.815	30.129	33.797	30.113
40 × 40	min	56.584	62.217	58.634	61.854	59.393	58.267	59.393	58.255	94.467	58.256
	std	6.258	6.989	4.985	5.260	7.655	7.601	6.541	5.530	13.927	8.827
	avg	64.589	70.055	65.315	69.629	65.767	65.424	67.651	67.247	111.415	68.618

## Data Availability

The raw data supporting the conclusions of this article will be available from the authors on request.
